# The Tetrahydrofuran Motif in Polyketide Marine Drugs

**DOI:** 10.3390/md20020120

**Published:** 2022-02-03

**Authors:** Laura Fernández-Peña, Carlos Díez-Poza, Paula González-Andrés, Asunción Barbero

**Affiliations:** Department of Organic Chemistry, Campus Miguel Delibes, University of Valladolid, 47011 Valladolid, Spain; laura.fernandez.pena@uva.es (L.F.-P.); carlos.diez@uva.es (C.D.-P.); paula.gonzalez.andres@alumnos.uva.es (P.G.-A.)

**Keywords:** marine natural products, oxygen heterocycles, tetrahydrofuran, total synthesis, biological activity

## Abstract

Oxygen heterocycles are units that are abundant in a great number of marine natural products. Among them, marine polyketides containing tetrahydrofuran rings have attracted great attention within the scientific community due to their challenging structures and promising biological activities. An overview of the most important marine tetrahydrofuran polyketides, with a focused discussion on their isolation, structure determination, approaches to their total synthesis, and biological studies is provided.

## 1. Introduction

The ocean is the biggest ecosystem of our planet. Plenty of organisms live in the ocean, and new species are discovered every year. As well, being the oldest ecosystem, marine organisms have evolved for a longer time than terrestrial living beings, and thus have different and sometimes better mechanisms of defense. These are represented by specific compounds, like toxins found in fish and algae and other bioactive substances found in sponges or tunicates. Usually, the real producers of these compounds are microorganisms such as bacteria, cyanobacteria, or dinoflagellates. The interesting properties of some of these metabolites have attracted the attention of the scientific community. As they are usually scarce and difficult to obtain in large amounts, total synthesis has emerged in the last decades as a necessary tool to tackle this problem. It has helped to clarify the structure of some intriguing compounds, and to obtain higher amounts of them in order to perform proper biological studies. Total synthesis is still a crucial tool, as even with today’s advanced NMR techniques, misassignment of the structure of biologically relevant macrolides is a common issue [1].

Polyketides are a diverse class of metabolites, comprising linear as well as macrolide compounds with a range of biological activities. Some of them have promising potential as drug candidates [2]. Common sources of this class of compounds are dinoflagellates of the genus *Amphidinium* [3]. To understand how to access the macrolide core present in these and other structures within natural products, we encourage the reader to review general macrolactonization methods [4].

On the other hand, oxygenated heterocycles are common motifs in marine bioactive compounds, and therefore the development of methods for their synthesis also requires attention. The most common are six-membered oxacycles, tetrahydropyrans, and thus numerous works focus on their synthesis [5,6]. Oxepanes and tetrahydrofurans also appear in a very large number of marine natural products with interesting properties. Oxepane-containing marine compounds were recently reviewed by our group [7]. Total syntheses of marine and non-marine products containing 2,3,5-trisubstituted tetrahydrofurans (such as kumausallene or petromyroxol) have been recently reviewed by Fernandes [8,9]. 

Continuing our interest for marine heterocyclic compounds, here we provide an overview, from 2013 to October 2021, of tetrahydrofuran-containing marine polyketides. The compilation of literature on THF-containing macrolides up to 2012 was covered in an excellent review [10]. Macrolides is such a fertile field that some particular families of compounds have already been reviewed, such as haterumalides and biselides [11], or more recently, mandelalides [12]. In recent years, numerous synthetic approaches to the polyketide family continue to emerge, and here we offer an overview of them.

## 2. Polyketide Marine Drugs Containing Tetrahydrofuran Rings

### 2.1. Macrolides

Marine invertebrates, such as sponges, algae or dinoflagellate, are a source of a large number of secondary metabolites with relevant biological activities. Within them marine polyketide macrolides have attracted high attention, due to their biological properties and pharmacological potential. Consequently, the development of new synthetic approaches to study their properties has been the goal of many researchers [10,13].

#### 2.1.1. Amphidinolides

Amphidinolides belong to the macrolide family and more than forty members have been isolated from marine dinoflagellates of the genus *Amphidinium* sp. In the last thirty years. Within these structurally rich compounds, only a few contain tetrahydrofuran units embedded in the macrolactone ring. The studies published in the literature about tetrahydrofuran-containing amphidinolides up to 2013 have been already reviewed by Álvarez and coworkers [10].

##### Amphidinolides C and F

**Figure 1:**
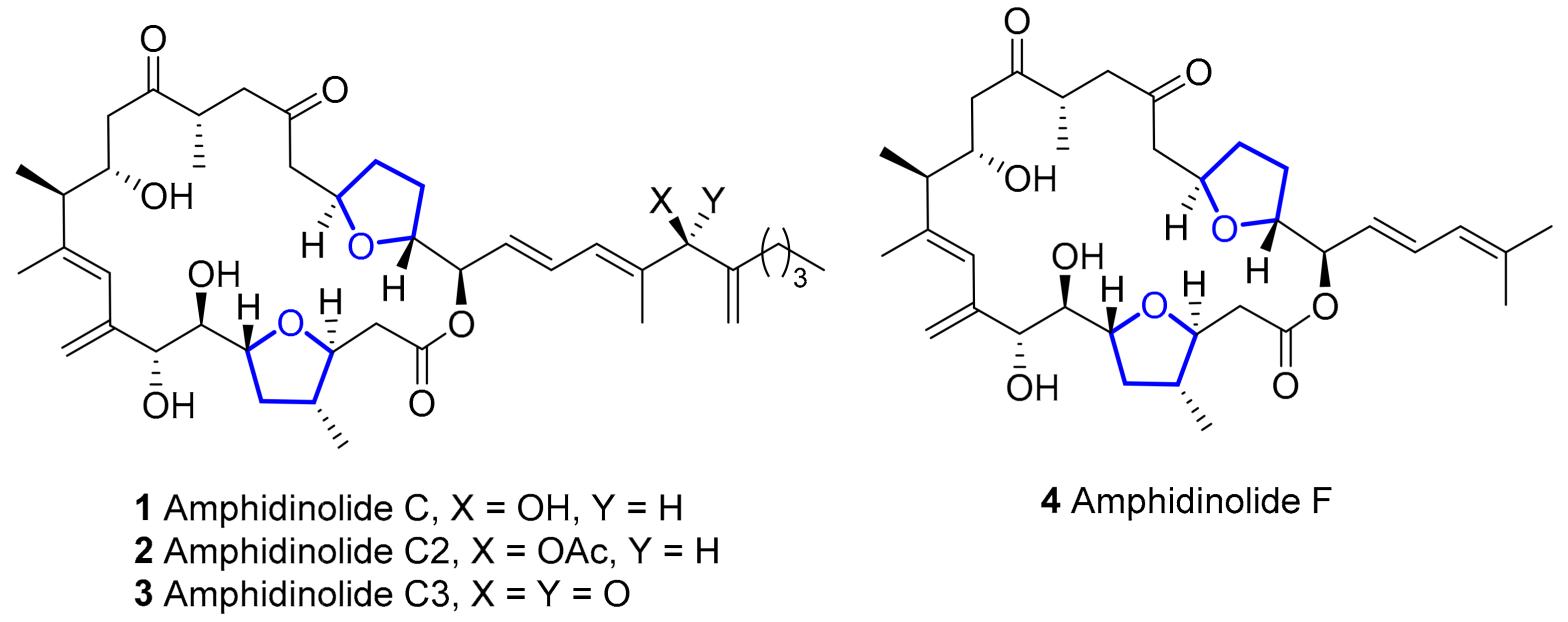
Structure of amphidinolides C, C2, C3, and F.

Amphidinolides C (**1**), C2 (**2**), C3 (**3**), and F (**4**) are structurally similar (Figure 1), bearing a complex 25-membered macrolide core, which contains 11 chiral carbons and a functionalized side chain. The nature of this chain drastically affects biological activity. Amphidinolide C, which possesses an (*S*)-hydroxyl group, is active against murine lymphoma L1210 (IC50 = 5.8 ng/mL) and human epidermoid carcinoma KB cell lines (IC50 = 4.6 ng/mL). Strikingly, compounds **2**-**4** are three orders of magnitude less toxic to the same cell lines. 

**Figure 2:**
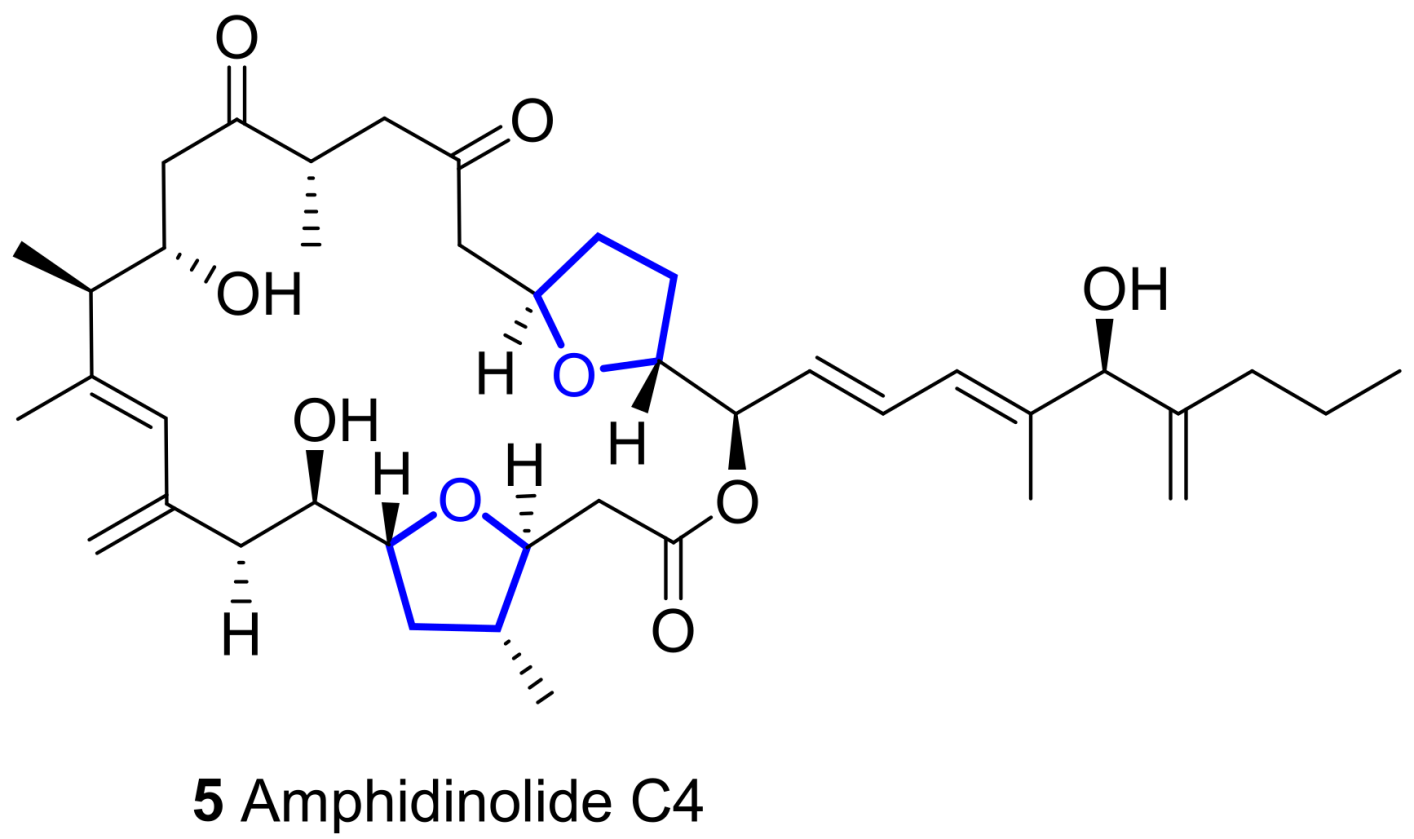
Structure of amphidinolide C4.

In 2016, a new amphidinolide C4 (**5**) was isolated from octocoral *Stragulum bicolor* (Figure 2). Toxicity was tested against the colon adenocarcinoma cell line HCT-116, finding an IC50 of 10.3 μM. Compound **5** is therefore surprisingly inactive, although it contains a hydroxyl group in the side chain [14].

**Scheme 1:**
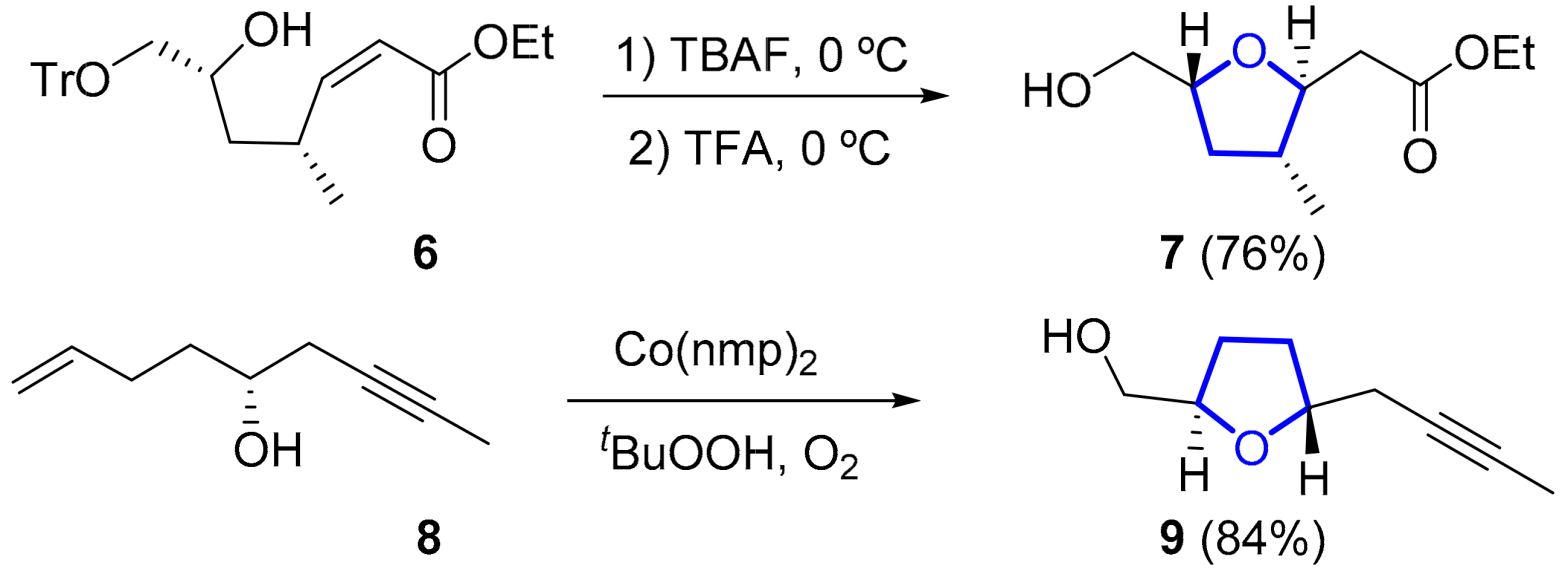
Synthesis of the THF moiety in amphidinolides C and F by Fürstner.

The interesting biological properties of these compounds have attracted the attention of different authors who have described either total syntheses [15,16] or synthetic approaches to various molecular fragments [17,18,19]. For instance, Fürstner and coworkers took advantage of the structural similarity of **1** and **4** to perform an elegant total synthesis of both compounds [15]. In these syntheses, the formation of the trisubstituted tetrahydrofuran unit was performed using a TBAF-mediated oxa-Michael addition, which selectively led to the desired 1,4-*trans* tetrahydrofuran ring **7**. Parallelly, a chemoselective cobalt-catalyzed cyclization of an appropriate bishomoallylic alcohol **8** provided the required *trans*-disubstituted tetrahydrofuran **9** (Scheme 1).

##### Amphidinolide E

**Figure 3:**
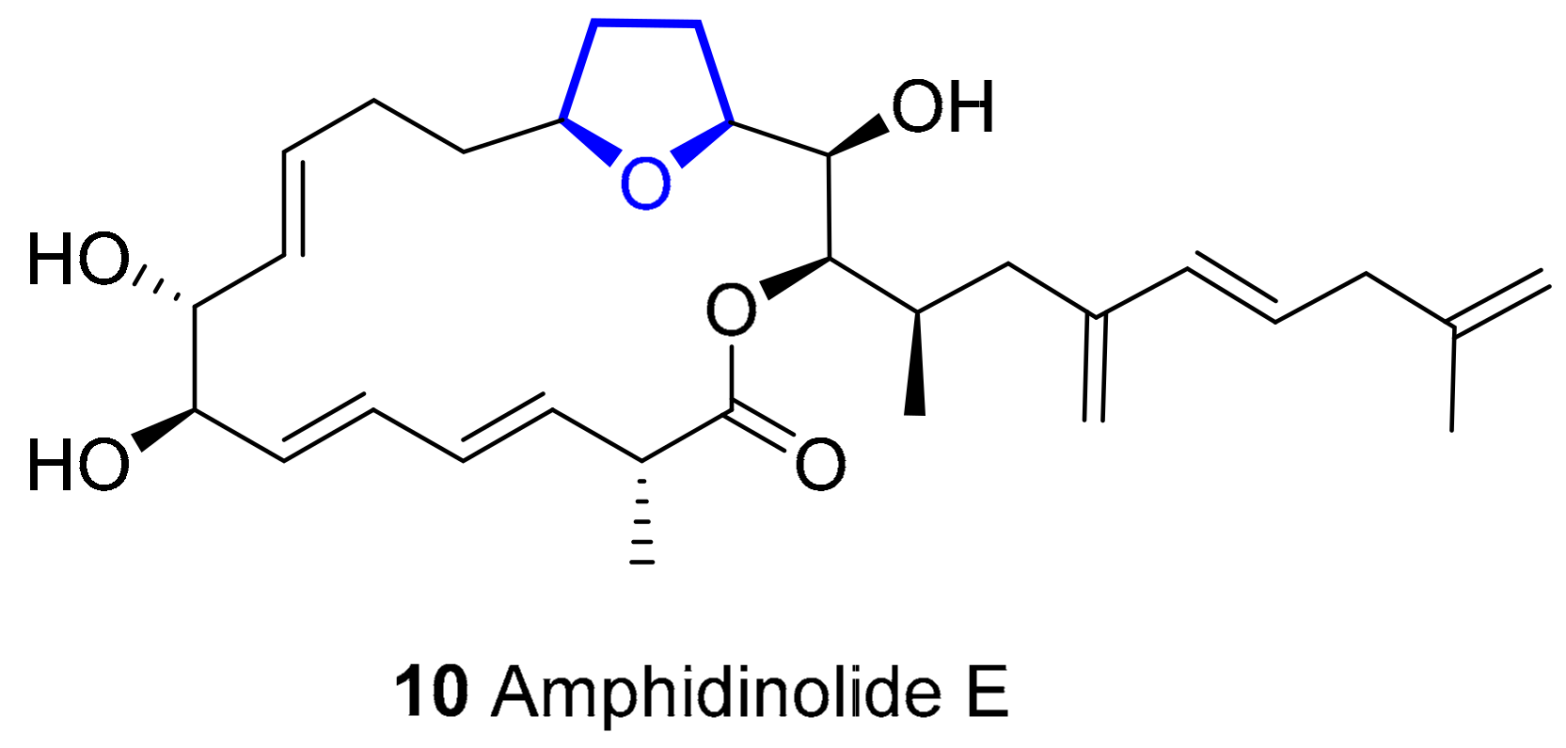
Structure of amphidinolide E.

Amphidinolide E (**10**) presents a 19-membered macrolactone ring, bearing a tetrahydrofuran moiety and eight stereocenters (Figure 3). 

**Scheme 2:**
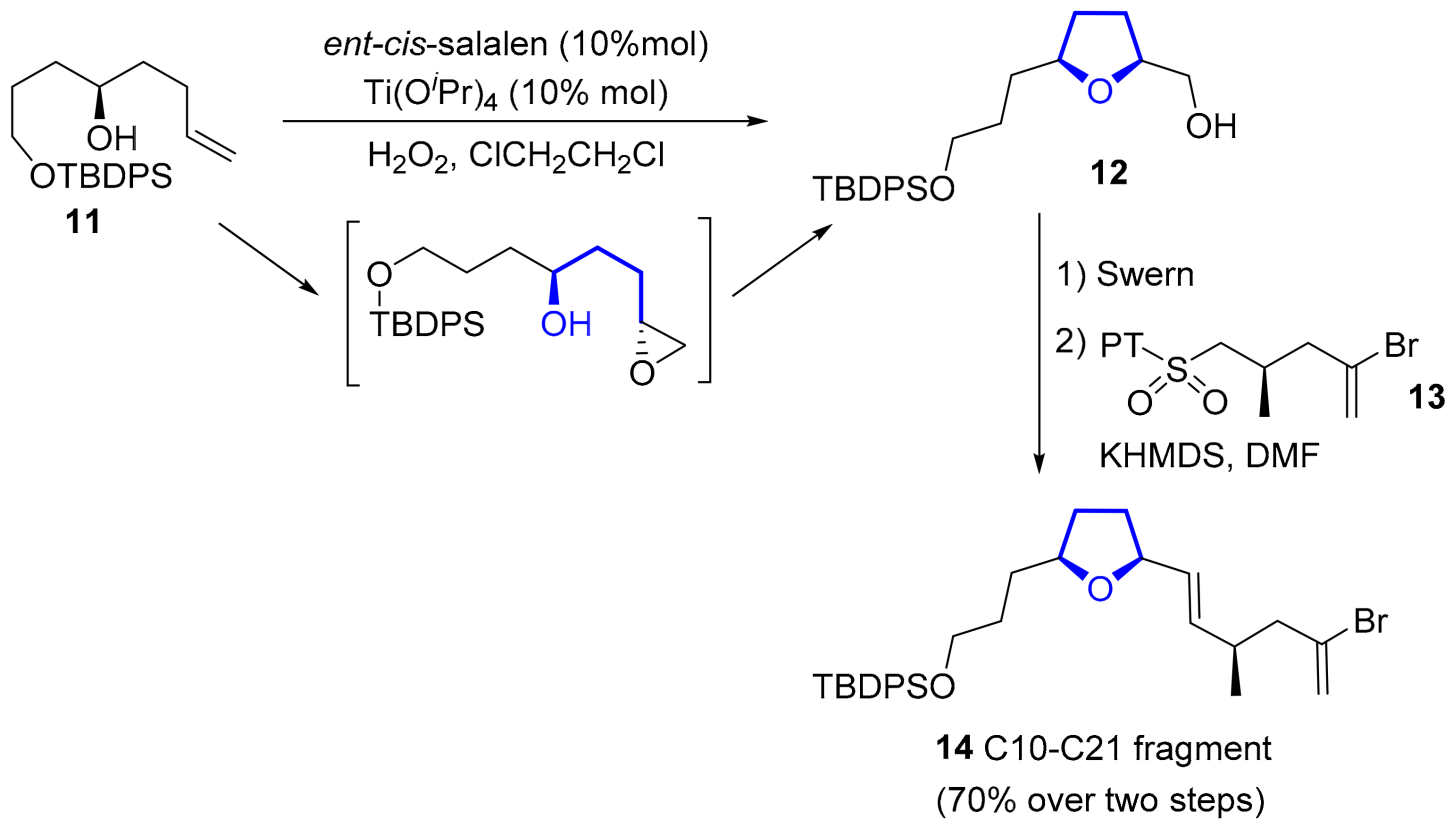
Synthesis of C10-C21 fragment of amphidinolide E.

Total syntheses of **10** have already been developed by Lee [20,21] and Roush [22,23,24]. Recently, Vilarrasa and Costa presented a different approach to its total synthesis. Though no significant improvement was made in terms of yield or number of steps, this work provides interesting insights into Julia-Kocienski olefinations [25]. Thus, epoxidation of alcohol **11** directly afforded tetrahydrofuran **12**, through a tandem epoxidation-cyclization reaction. Subsequent Swern oxidation, followed by Julia-Kocienski reaction with sulfone **13**, provided the northern C10–C21 fragment of amphidinolide E (Scheme 2).

##### Amphidinolide K

**Figure 4:**
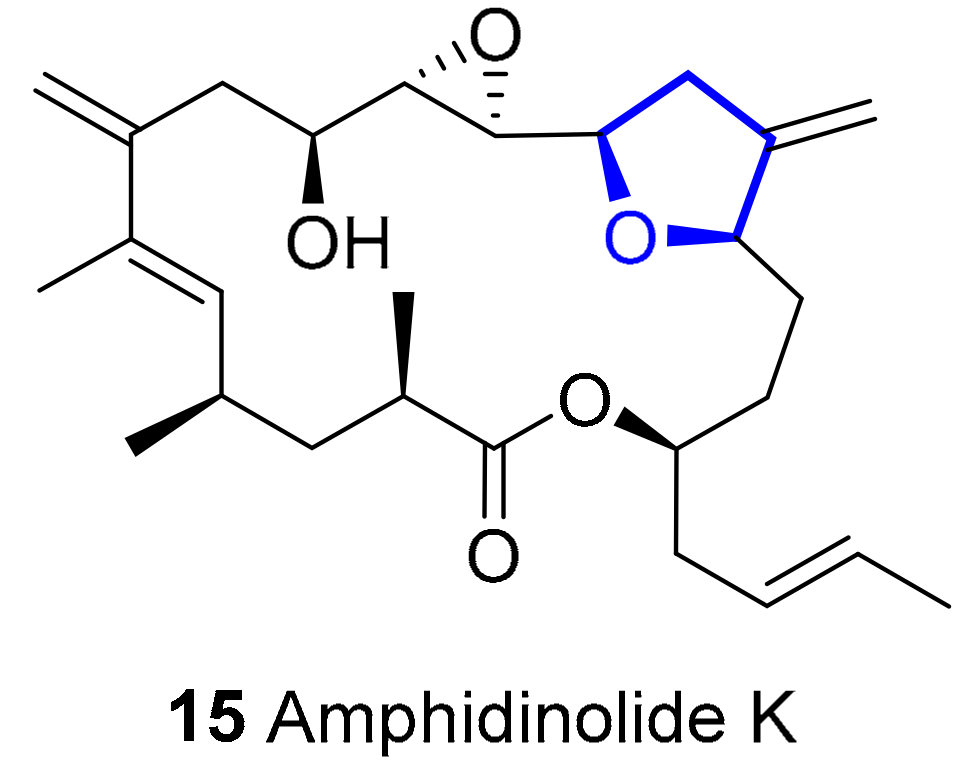
Structure of amphidinolide K.

Amphidinolide K (**15**) (Figure 4) has also a 19-membered macrolactone, but with a simpler side chain. Regarding its biological properties, amphidinolide K has shown a strong stabilizing effect on actin filaments (F-actin).

**Scheme 3:**
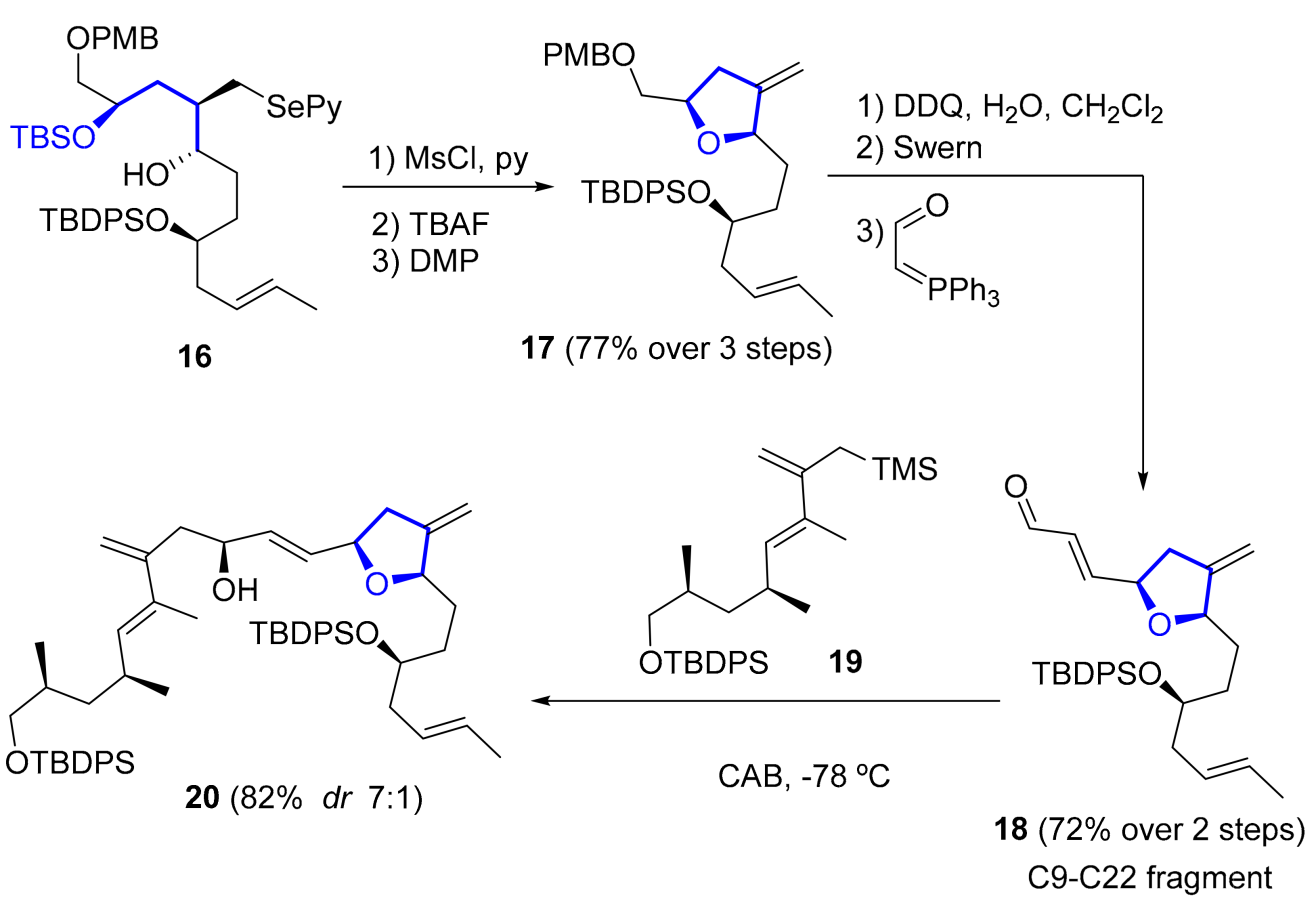
Synthesis of C9–C22 fragment of amphidinolide K.

Vilarrasa proposed a total synthesis of **15** relying on a Hosomi-Sakurai reaction as a key step [26]. The tetrahydrofuran ring **17** was formed by cyclization of alcohol **16**, followed by elimination of the pyridylselenenyl group with Dess-Martin oxidation. Further deprotection of the O-PMB group and extension of the chain, through Swern oxidation and Wittig reaction, led to the C9–C22 fragment **18** (Scheme 3). Subsequent Hosomi-Sakurai reaction with allylsilane **19**, using Yamamoto’s chiral (acyloxy)borane (CAB), provided **20** with a good yield.

##### Amphidinolide N/caribenolide I

**Figure 5:**
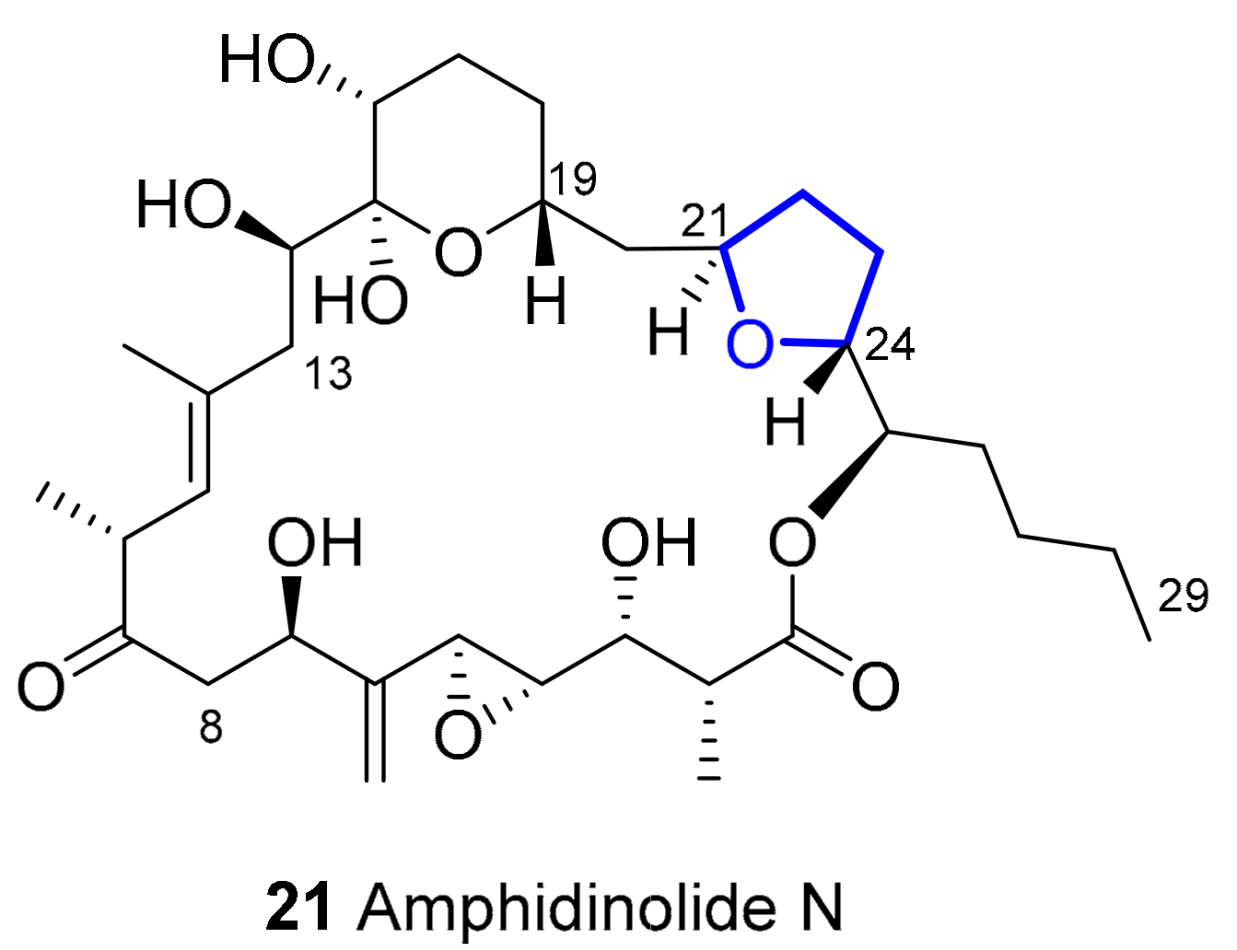
Structure of amphidinolide N.

Amphidinolide N (**21**) is a 25-membered macrolide which contains a 2,5-*trans*-disubstituted tetrahydrofuran, an allylic epoxide, and 13 stereocenters (Figure 5). Amphidinolide N is the most potent cytotoxic member of this family against murine lymphoma L1210 (IC50 = 0.05 ng/mL) and human epidermoid carcinoma KB cell lines (IC50 = 0.06 ng/mL). However, despite the efforts of different researchers, no total synthesis of this compound has been reported so far. There are different approaches focused on the synthesis of the fragment which contains the tetrahydrofuran ring [27,28,29,30], a total synthesis of 7,10-epimer [31,32], and a recently described enantioselective synthesis of des-epoxy-amphidinolide N, which failed in the last epoxidation step towards amphidinolide N [33]. 

**Scheme 4:**
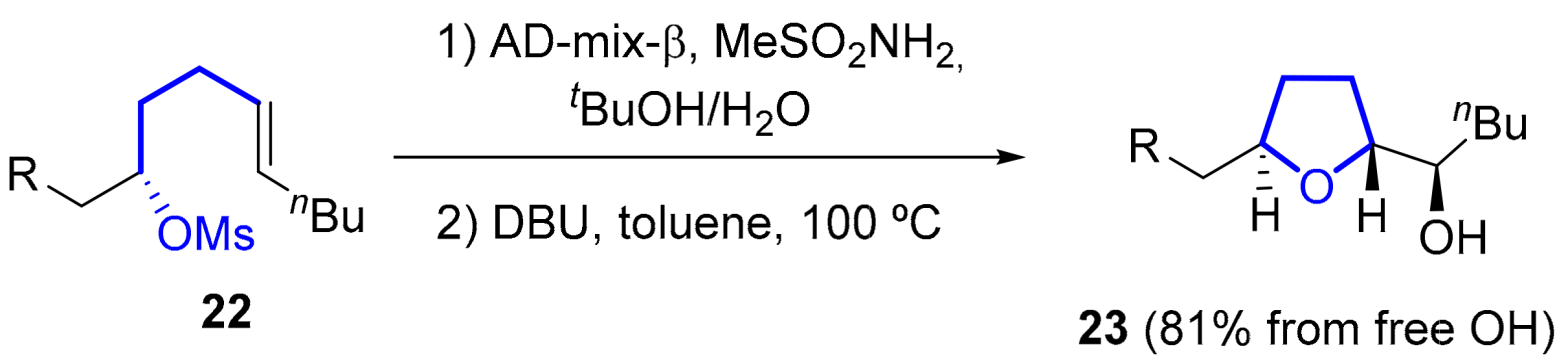
Synthesis of THF of amphidinolide N by Sasaki and Kuwahara.

Both Sasaki’s [27] and Kuwahara’s [29] strategies to build the tetrahydrofuran moiety consisted of an intramolecular cyclization of a diol mesylate obtained by Sharpless asymmetric dihydroxylation (SAD) of mesyl protected alkenol **22** using AD-mix-β. Subsequent cyclization, mediated by base, afforded the desired 2,5-*trans-*disubstituted tetrahydrofuran **23** (Scheme 4). 

**Scheme 5:**
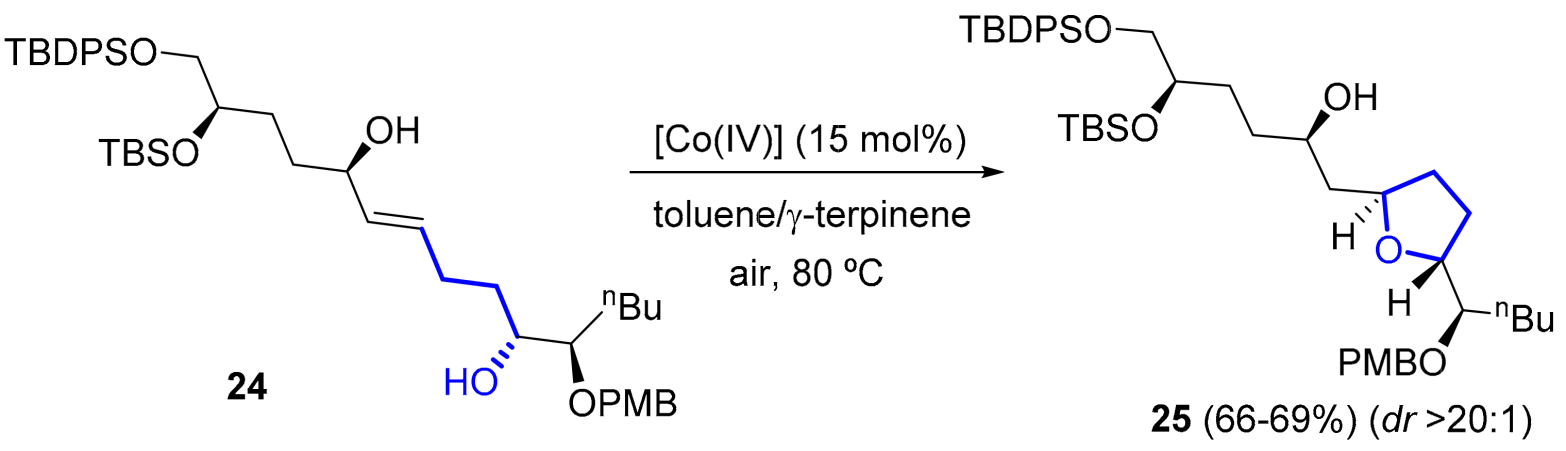
Synthesis of THF fragment of amphidinolide N by Fuwa.

Recently, Fuwa proposed the application of cobalt-catalyzed Hartung-Mukaiyama cyclization of γ-hydroxy olefins to obtain the tetrahydrofuran fragment [30]. Mukaiyama cyclization is known to afford 2,5-*trans* substituted tetrahydrofurans with a 2-hydroxy substituent. Hartung’s modification allows the access to 2-alkyl-substituted tetrahydrofurans, ideal for the synthesis of tetrahydrofuran-containing fragment **25** of amphidinolide N from alkenol **24** (Scheme 5). It is remarkable that unprotected hydroxy groups and somewhat bulky substituents are well-tolerated.

**Figure 6:**
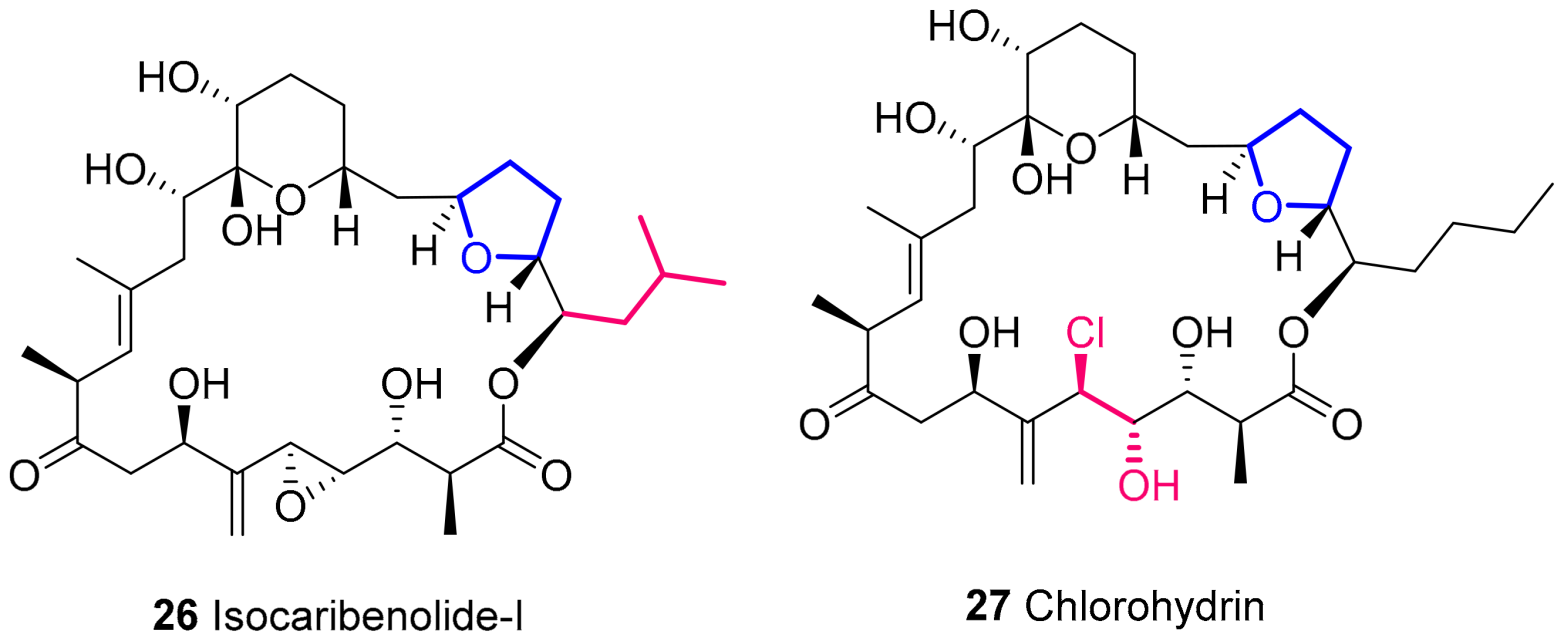
Structure of isocaribenolide I and chlorohydrin.

With close structural similarity, other macrolides have been discovered recently, namely isocaribenolide-I (**26**) and chlorohydrin (**27**) [34]. They were isolated from a free-swimming dinoflagellate Amphidinium species (KCA09053 and KCA09056 strains), together with amphidinolide N. Both have a 26-membered macrolide core, and present high cytotoxicity against human cervix adenocarcinoma HeLa cells (IC50 = 0.02 for isocaribenolide-I and 0.06 nM for chlorohydrin). Isocaribenolide-I presents a characteristic isobutyl side chain, and chlorohydrin is distinguished by a homonymous moiety (Figure 6).

##### Amphidinolides T

**Figure 7:**
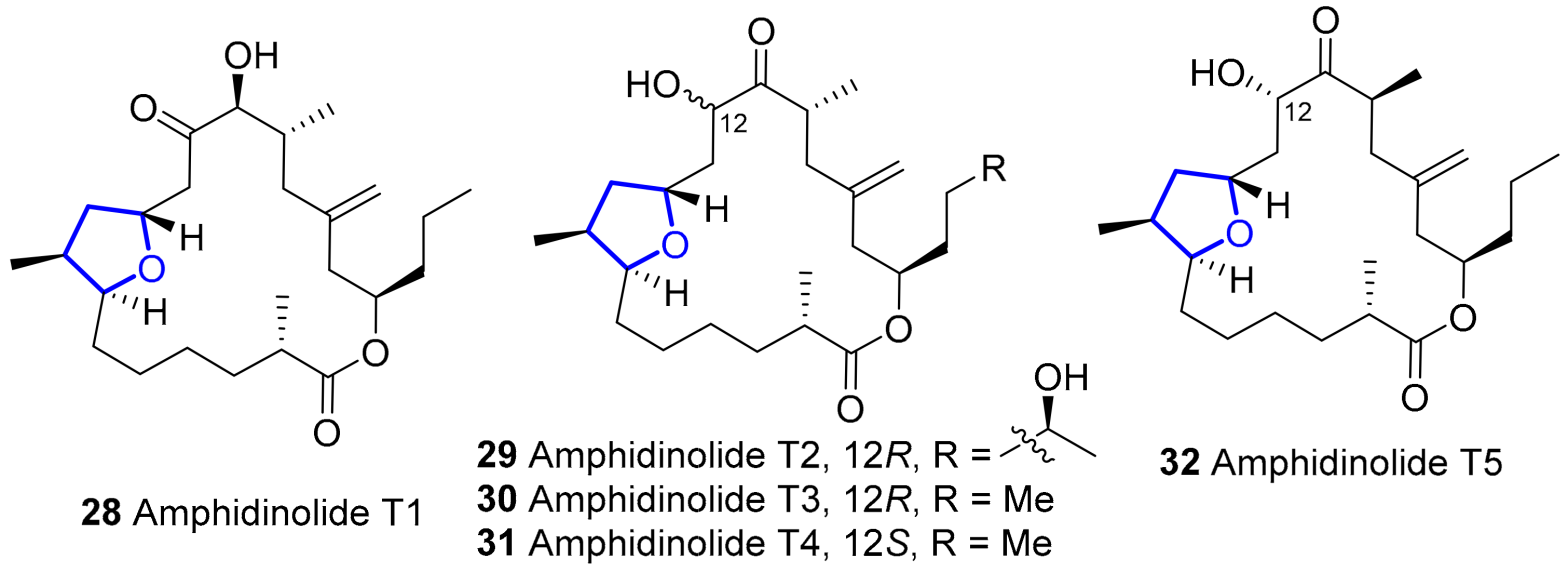
Structure of amphidinolides T.

Amphidinolides T (**28**–**32**), 19-membered lactones containing a trisubstituted tetrahydrofuran ring and seven or eight stereocenters (Figure 7), were first isolated by Kobayashi [35,36,37]. They showed cytotoxic activity against murine leukemia L1210 cells in vitro with an IC50 value of 18 μg/mL. 

**Scheme 6:**
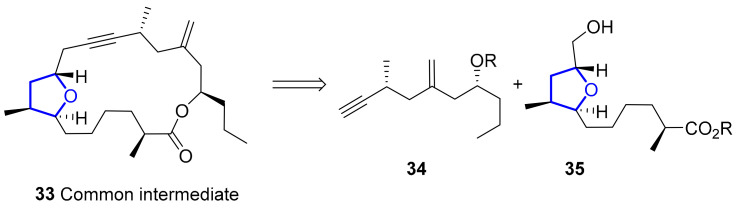
Retrosynthesis of amphidinolides T from a common intermediate.

**Scheme 7:**
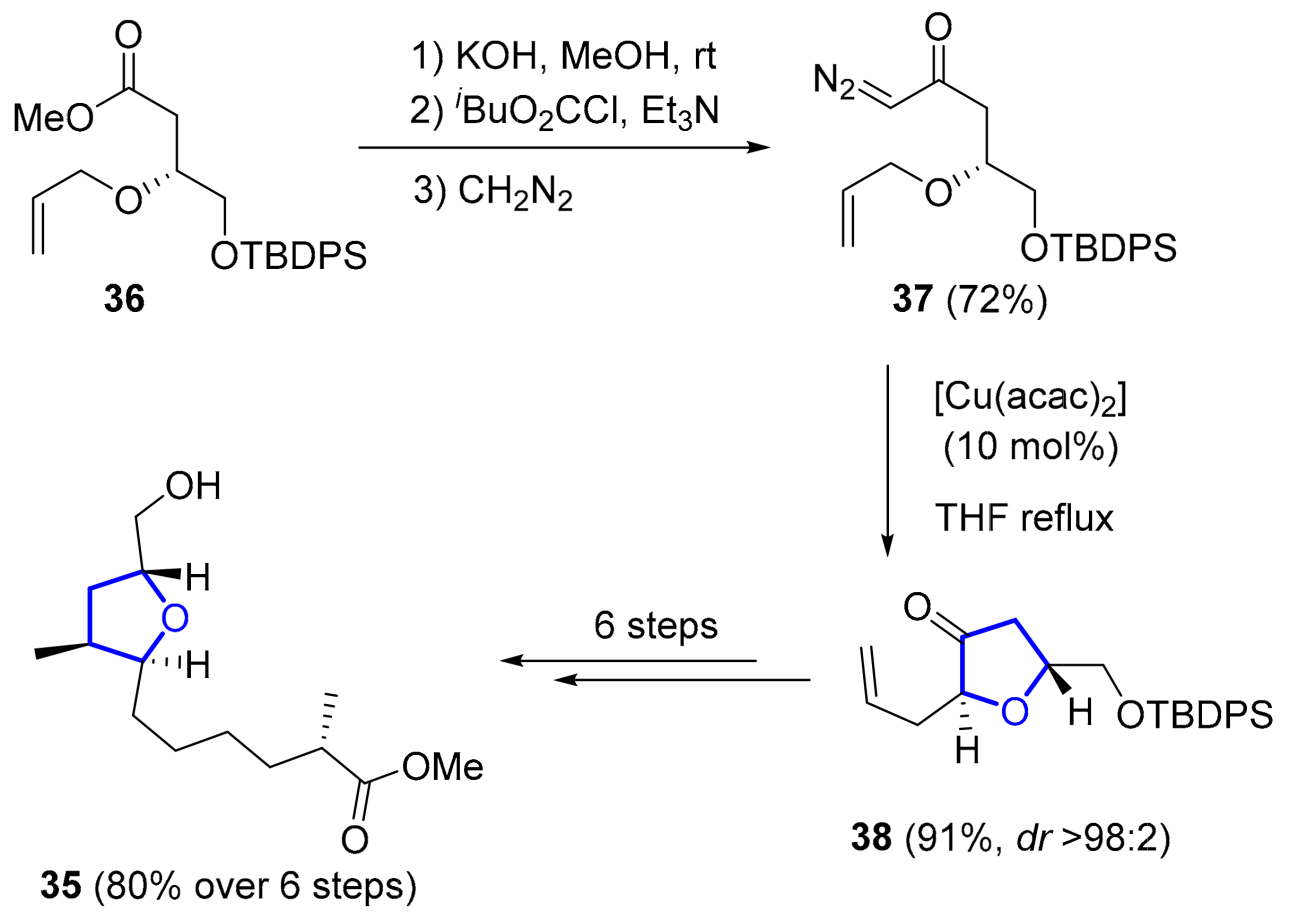
Synthesis of THF fragment of amphidinolides T.

In 2013, Clark reported a total synthesis of T1, T2, and T4 from a common intermediate **33** (Scheme 6) [38]. Synthesis of the tetrahydrofuran-containing fragment 35 started from the allyl ether **36** of a commercially available alcohol, which was transformed into the α-diazo ketone **37** by sequential saponification, formation of the corresponding mixed anhydride derivative and reaction with diazomethane. Treatment of **37** with a catalytic amount of (Cu(acac)_2_) stereoselectively afforded the *trans* dihydrofuranone **38** in high yield. Six further steps led to the desired THF-containing fragment (Scheme 7). The total syntheses of amphidinolide T1, T3, and T4 were completed in 17 steps from a common precursor with 6.9%, 5.9%, and 5.5% overall yield, respectively.

#### 2.1.2. Haterumalides and Biselides

**Figure 8:**
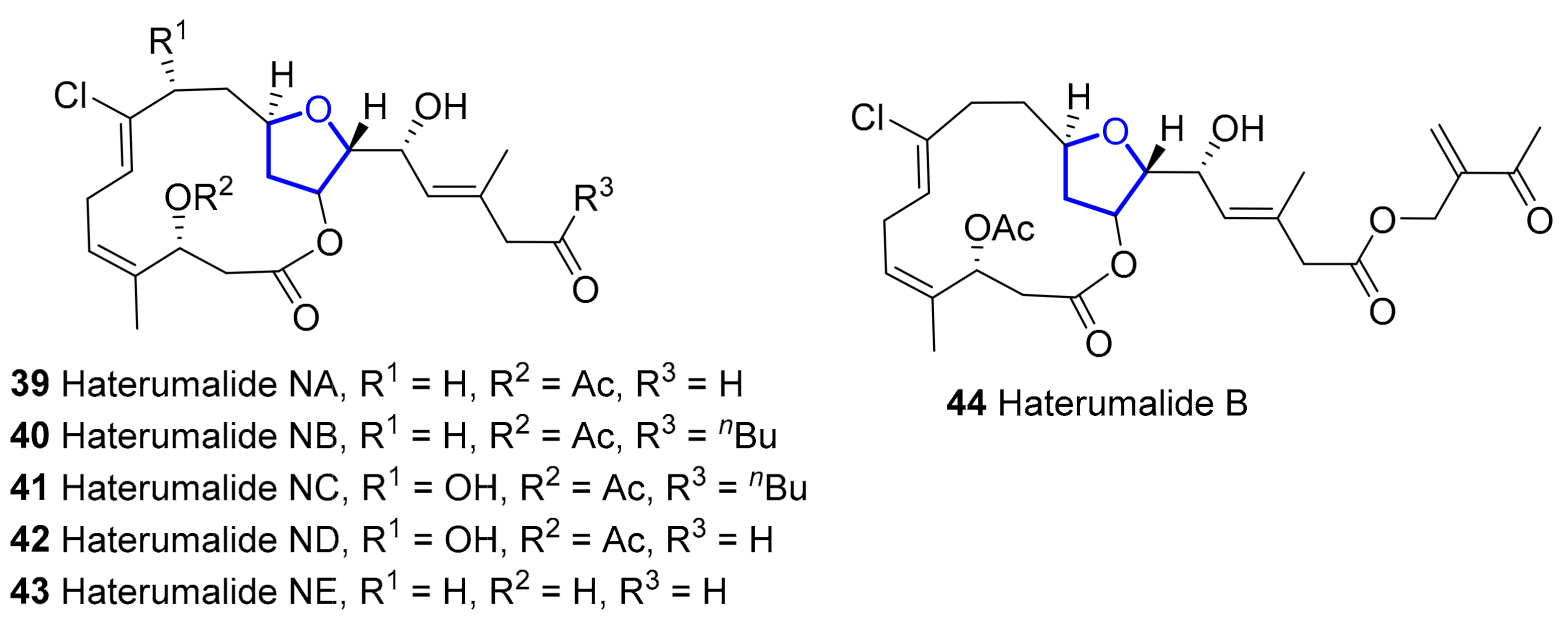
Structure of haterumalides.

Haterumalides (**39**–**44**) (Figure 8) were isolated at the end of the 20th century from the Okinawan ascidian *Lissoclinum* sp. (haterumalide B) [39] and the Okinawan sponge *Iricinia* sp. (haterumalides NA-NE) [40]. Their cytotoxic activity against different targets made them secondary metabolites of great interest. However, no approaches towards their synthesis have been described in recent years [10,11].

**Figure 9:**
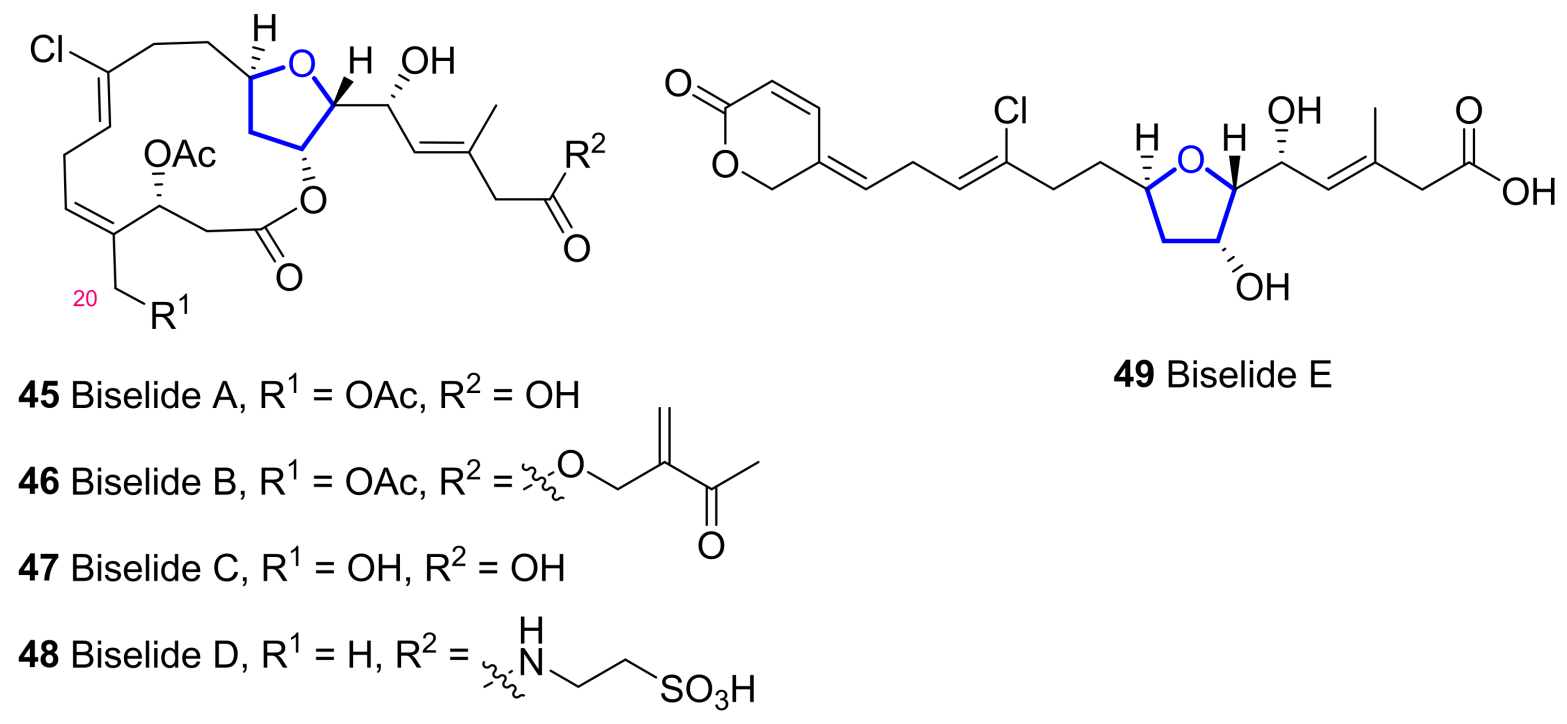
Structure of biselides.

Biselides (**45**–**49**) (Figure 9), C-20 oxygenated analogues of haterumalides, are a family of polyketides which were isolated from the Okinawan ascidian *Didemnidae* sp. [41]. Their structure was determined by spectroscopic analysis and their biological activity was tested against tumor cell lines, due to their similarity to haterumalides. Cytotoxic activity of biselides A and C against various human cancer cells are comparable to cisplatin, the known anticancer drug. Notably, and unlike their haterumalide congeners, biselides A and C did not show toxicity against brine shrimp even at 50 μg mL^−1^, which makes them potential anticancer drug candidates.

**Scheme 8:**
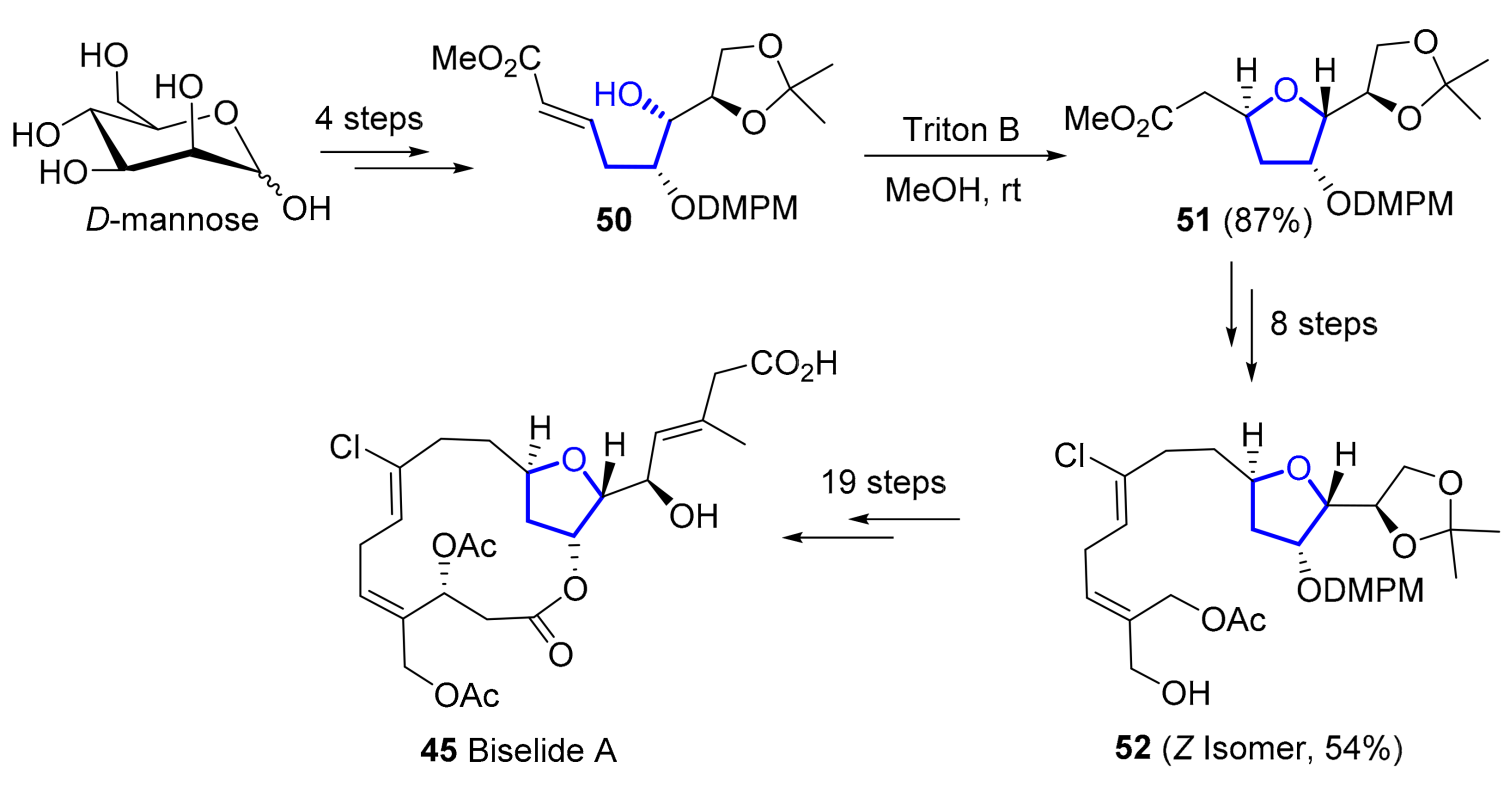
Synthesis of biselide A by Kigoshi, Hayakawa, and co-workers.

In the last years, two total syntheses of biselide A have been published [42,43]. Kigoshi developed synthetic approaches towards both the core carbon fragment [44] and the macrolactone moiety [45]. Relying on these methodologies, Kigoshi and Hayakawa finally accomplished the total synthesis of biselide A [42]. The key step for the construction of the 3-hydroxy tetrahydrofuran unit was accomplished by intramolecular oxy-Michael cyclization of intermediate **50**, which was obtained in four steps from D-mannose. Further 27 steps were required to obtain natural biselide A (Scheme 8).

Likewise, Kigoshi has also described the total synthesis of biselide E, from common advanced intermediate **52** [46].

**Scheme 9:**
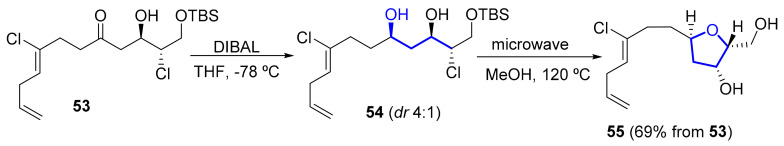
Synthesis of THF moiety of biselide A in Britton’s total synthesis.

On the other hand, Britton and coworkers have proposed a concise synthesis of biselide A in 20 linear steps from L-serine [43]. In this case the stereoselective formation of the tetrahydrofuran moiety **55** was done by microwave cyclization of chlorodiol intermediate **54** (Scheme 9).

**Scheme 10:**
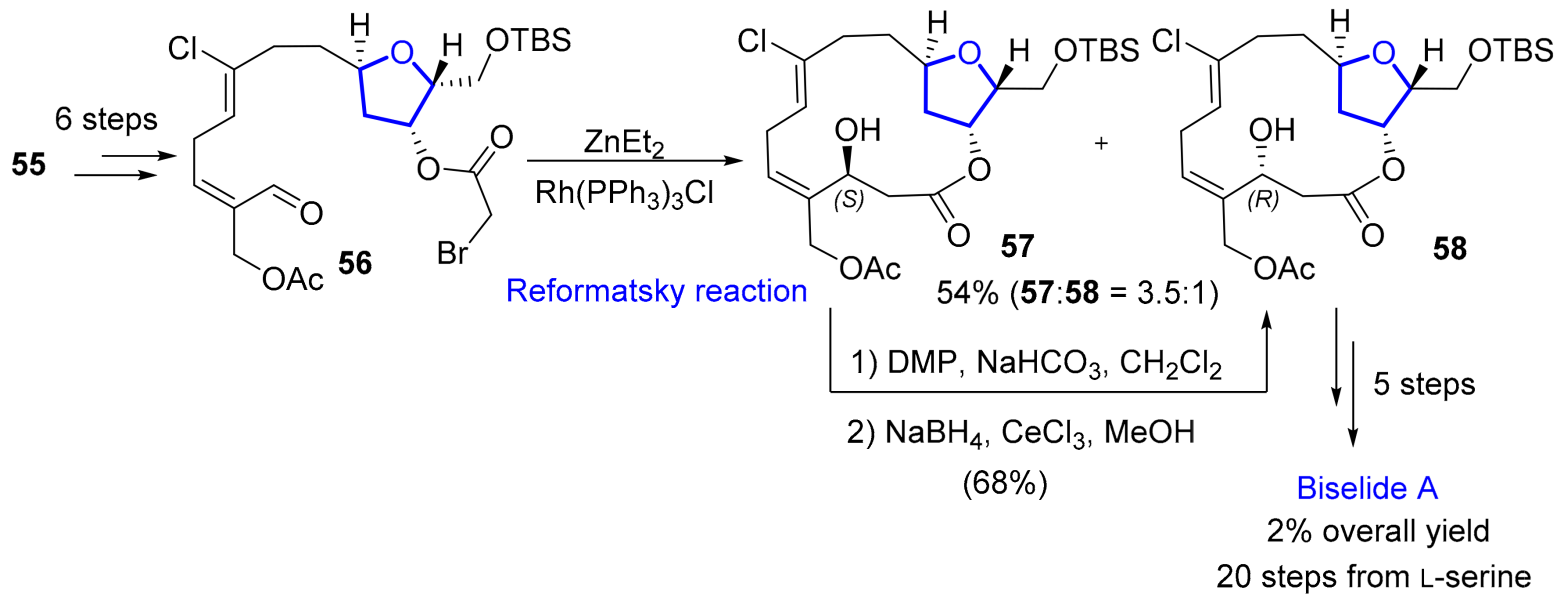
Reformatsky macrocyclization to biselide A by Britton and coworkers.

The key to building the macrocyclic ring was the use of an intramolecular Reformatsky cyclization from intermediate **56**, which led to a 3.5:1 ratio of epimers of the desired macrolactone. A sequence of oxidation/reduction was used to convert **57** in the desired epimer **58**. Further five steps, including deprotection and installation of the side chain, provided biselide A with ca. 2% overall yield in 20 steps from L-serine (Scheme 10).

#### 2.1.3. Chagosensine

**Figure 10:**
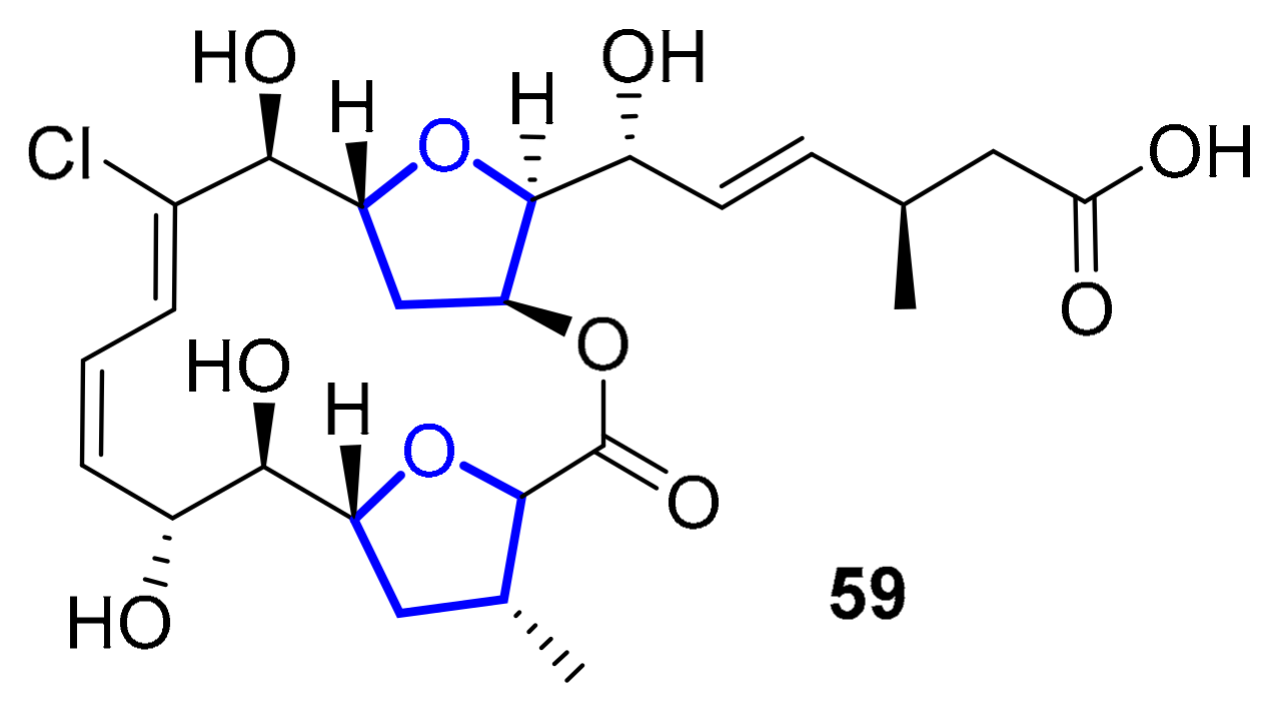
Initially proposed structure of chagosensine.

Chagosensine (**59**) (Figure 10), a chloro-substituted macrolide, was isolated from the calcareous sponge *Leucetta chagosensis*. Its structure, consisting of a sixteen-membered macrolide, two 2,5-*trans*-disubstituted tetrahydrofuran rings embedded within, a unique *Z,Z*-configured chloro-1,3-diene unit, and eleven chiral centers, was assigned by spectroscopic techniques and degradation experiments [47]. Chagosensine structure is similar to the haterumalide and biselide families, but the conjugated chlorodiene unit is only present in this natural product. Nevertheless, Fürstner and co-workers have shown disagreement with the proposed structure and have carried out different synthetic studies in order to demonstrate their arguments [48,49].

**Scheme 11:**
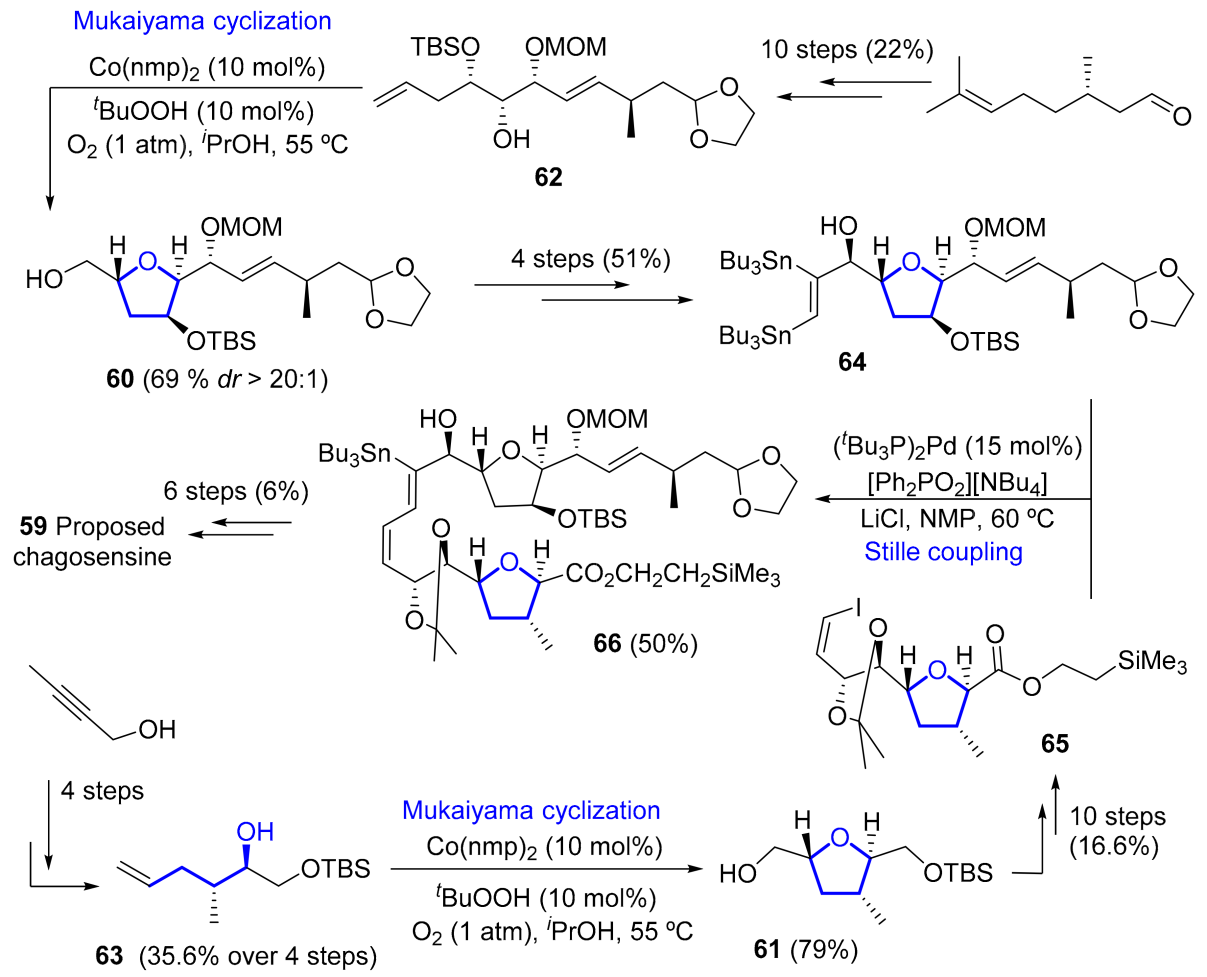
Synthesis of putative chagosensine.

The synthesis of putative chagosensine proposed by Fürstner relied on Mukaiyama cyclizations using Co(II) catalyst to obtain 2,5-*trans*-tetrahydrofuran derivatives **60** and **61** from the appropriate alkenols **62** and **63** [48]. After further elaboration, optimized Stille coupling of 1,2-bisstannane derivative **64** and vinyl iodide **65** provided the precursor diene **66** in moderate yield. Finally, six additional steps, including a Yamaguchi lactonization, produced the desired macrocycle (Scheme 11). The product proved unstable and had to be transformed into the known methyl esther, though huge deviations in the NMR seemed to indicate that the structure was misassigned. More recently, further efforts were made to access eight different diastereomers in order to find the correct structure [49]. Unfortunately, none of the structures synthesized matched the original spectroscopic data. Thus, the structure of this intriguing marine macrolide remains unresolved.

#### 2.1.4. Formosalides

**Figure 11:**
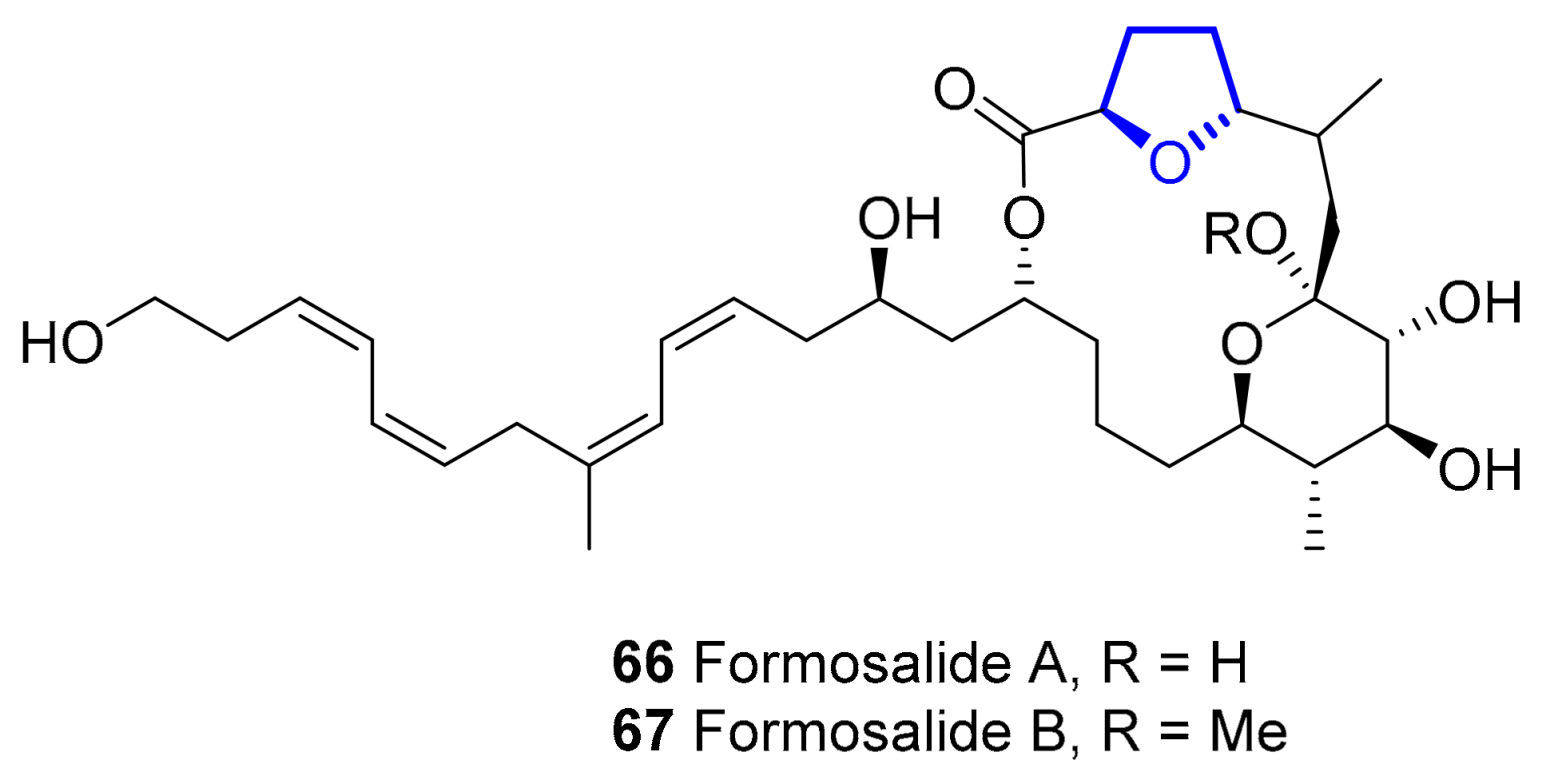
Structure of formosalides A and B.

Formosalides A and B (Figure 11) are 17-membered macrolides isolated from marine dinoflagellate *Prorocentrum* sp. [50]. Their structures and relative stereochemistries were determined by spectroscopic techniques. Cytotoxicty of the formosalides was significantly lower than amphidinolide N or caribenolide I, despite their structural similarity. Formosalides A and B showed in vitro moderate cytotoxic activity against CCRF-CEM human T-cell acute lymphoblastic leukemia cells (LD50 [A] = 0.54 μg/mL and LD50 [B] = 0.43 μg/mL) and DLD-1 human colon adenocarcinoma cells (LD50 [A] > 40 μg/mL and LD50 [B] = 2.73 μg/mL) [50]. The structure and absolute configuration were confirmed by Fürstner and co-workers recently by total synthesis of different stereoisomers [51].

**Scheme 12:**
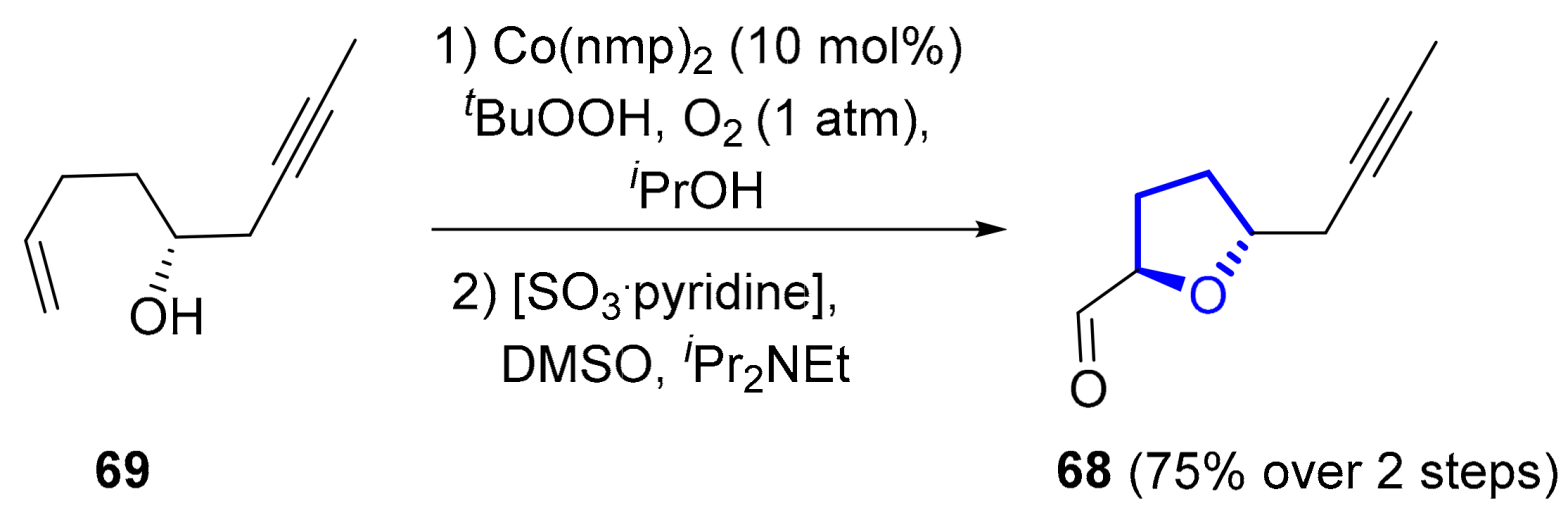
Synthesis of THF in formosalides.

As said, Fürstner’s group has accomplished the synthesis of both macrolides and two other isomers [51]. The required *trans*-disubstituted tetrahydrofuran ring **68** was prepared by stereoselective cobalt-catalyzed oxidative cyclization of bishomoallylic alcohol **69**, with excellent yield and selectivity (Scheme 12).

**Scheme 13:**
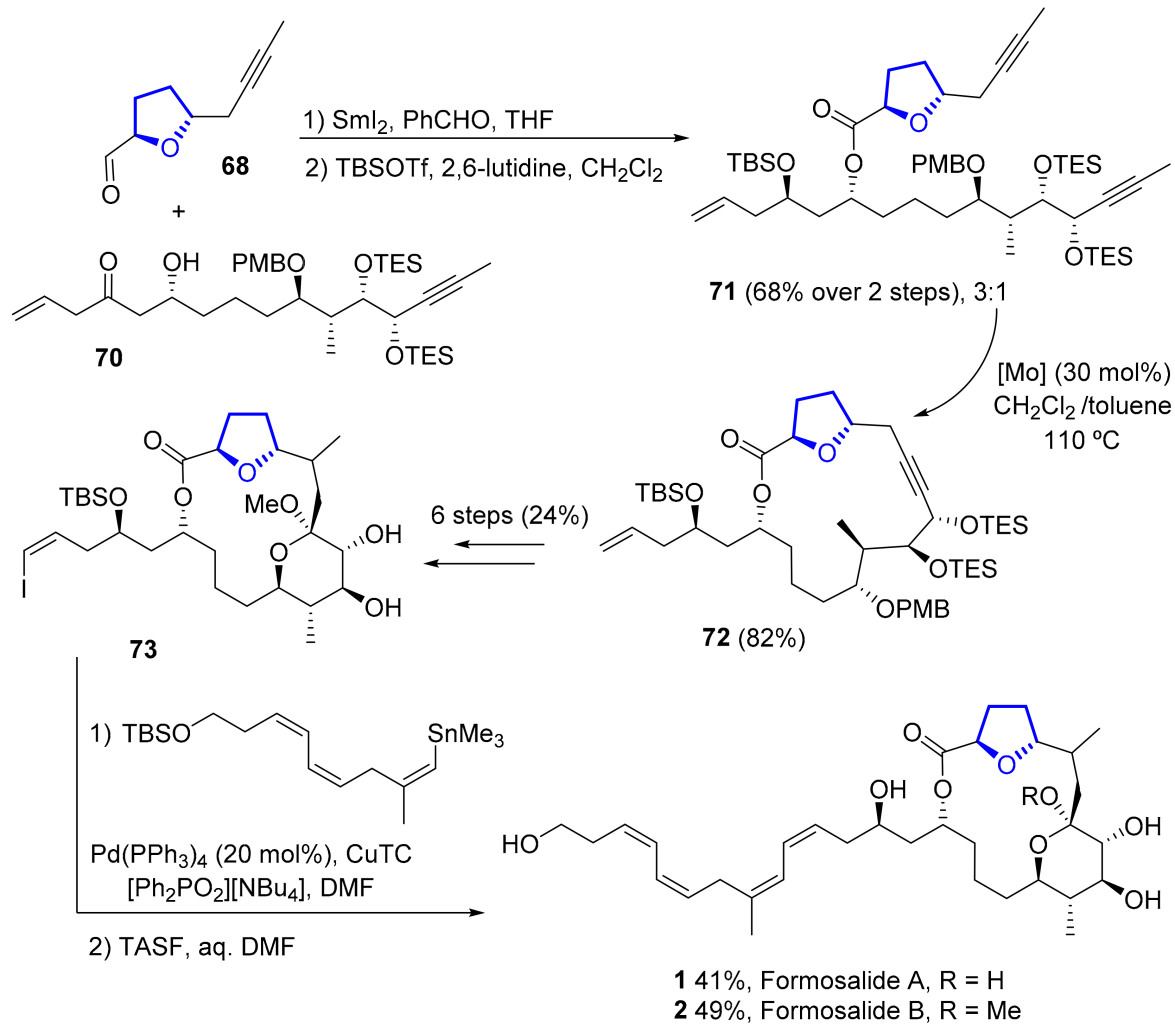
Synthesis of formosalides by Fürstner.

Following, the coupling of THF-carbaldehyde **68** with fragment **70** by an Evans–Tishchenko reaction led to intermediate **71**, which was subjected to ring closing alkyne metathesis to yield macrocycle **72** with good yield. The final installation of the side chain, achieved by Stille-Migita coupling of vinyliodide **73** with the appropriate vinylstannane derivative, and deprotection of the TBS group led to both formosalides due to unexpected partial hydrolysis of the ketal (Scheme 13). 

**Scheme 14:**
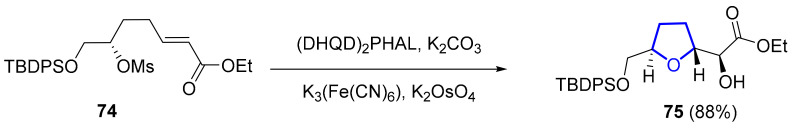
Synthesis of THF-containing fragment in formosalides by Mohapatra and co-workers.

Mohapatra and co-workers have also reported a stereoselective synthesis of the C1-C16 fragment of formosalide B [52]. A one-pot Sharpless asymmetric dihydroxylation of α,β-unsaturated ester **74**, followed by intramolecular S*_N_*2 displacement, produced the desired *trans*-tetrahydrofuran **75** with a high 88% yield and total stereoselectivity (Scheme 14).

#### 2.1.5. Halichondrins

**Figure 12:**
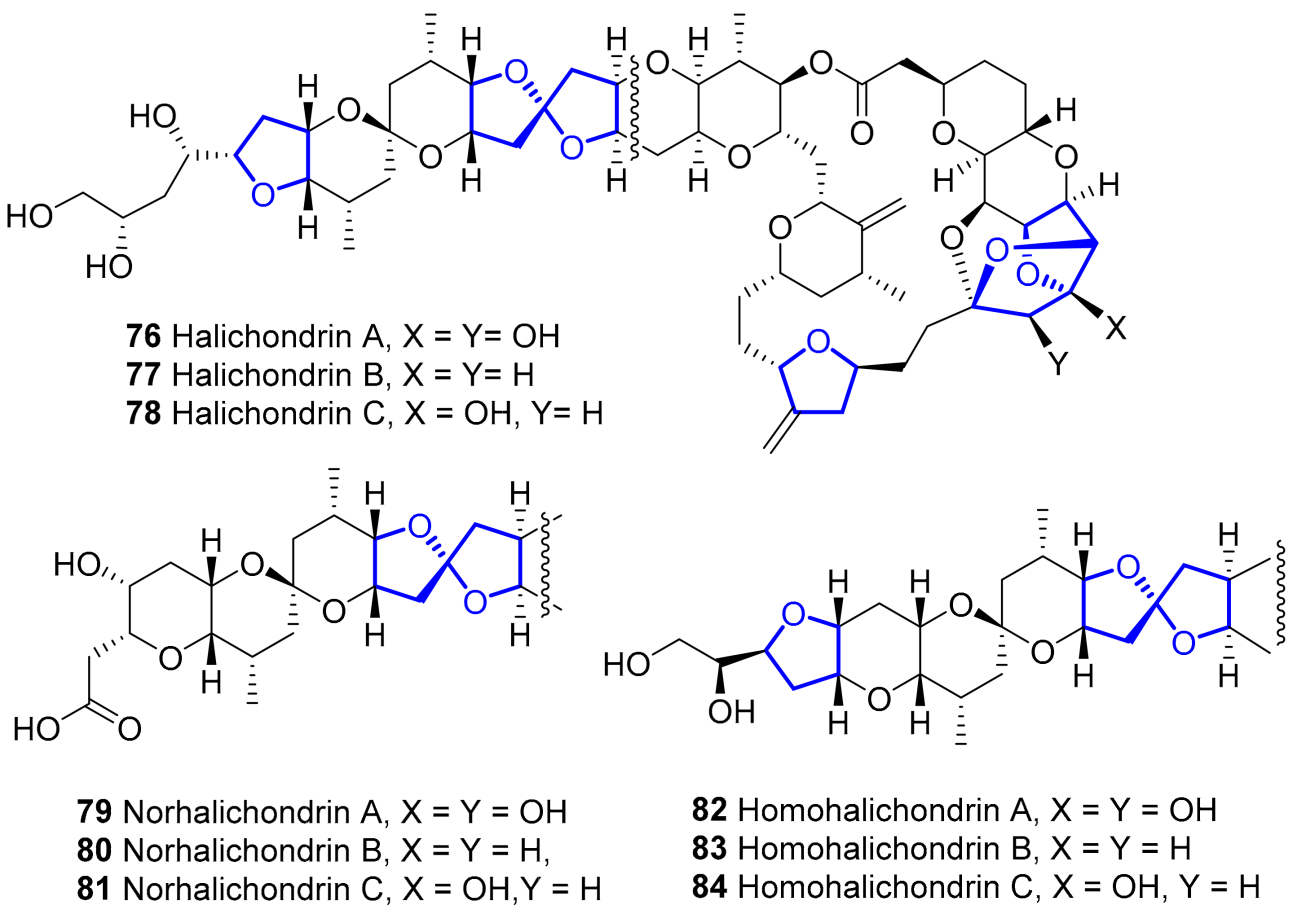
Structure of halichondrins, norhalichondrins and homohalichondrins.

Halichondrins (**76**–**84**) (Figure 12) are a family of polyether macrolides isolated in the 20th century from a marine sponge, *Halichondria okadai* Kadota [53]. According to their structure, they are classified in A–C halichondrins, norhalichondrins, and homohalichondrins (Figure 12). All have been found in natural sources except halichondrin A. Their complex structure is composed of a polyether macrolide with several fused five- and six-membered oxacycles.

**Scheme 15:**
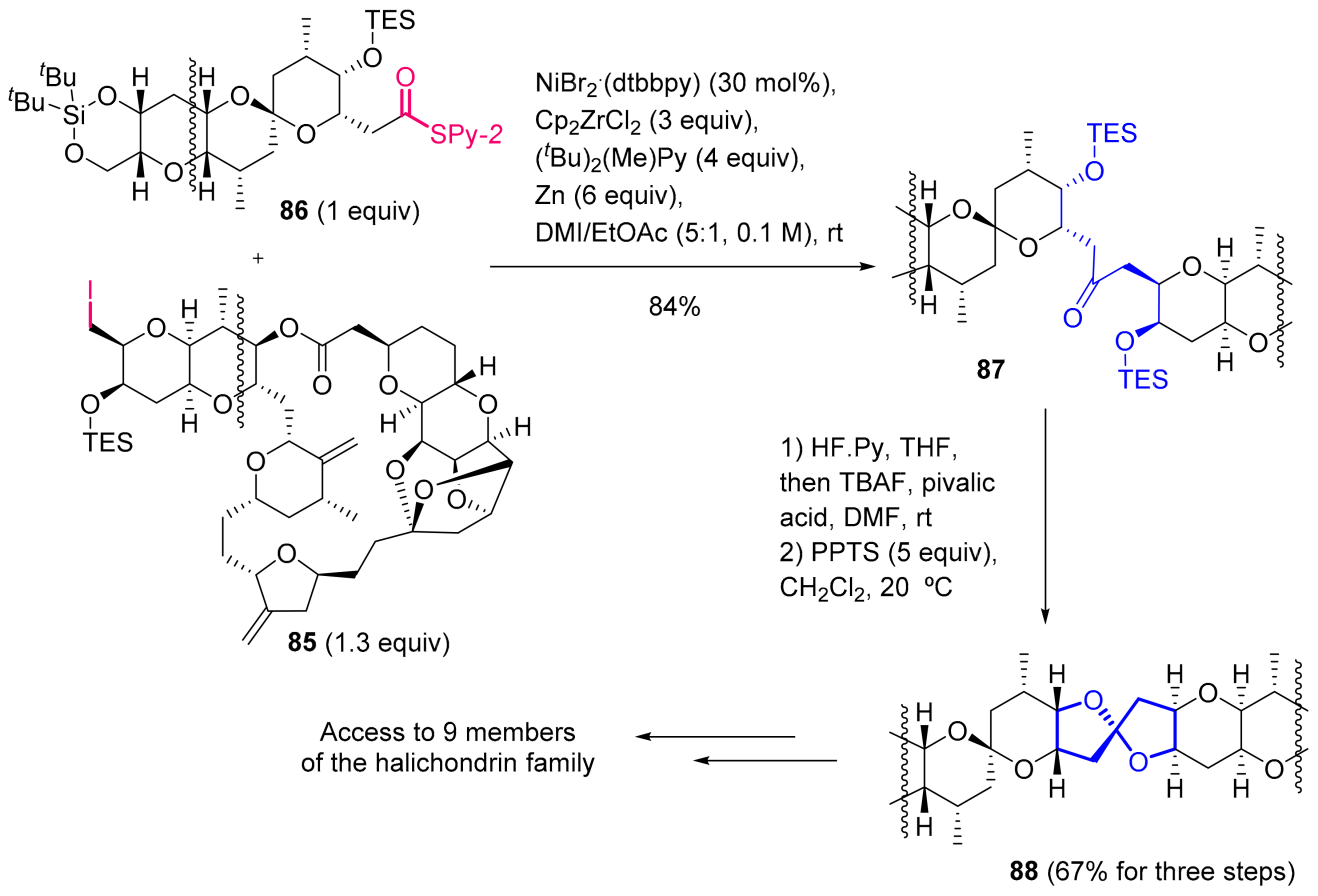
Key step in the total synthesis of halichondrins by Kishi.

Great efforts by Kishi’s group have been devoted to the synthesis of this family of compounds. To finally reach the total synthesis, Kishi et al. first established the synthesis of different fragments of halichondrins such as: C1–C19, through key Ni/Cr-mediated coupling of polyhalogenated nucleophiles [54,55], C14–C38 fragment, by oxy-Michael cyclization [56,57], and those combined led to the C1–C37 right halves of halichondrins A-C [58]. In 2017, Kishi and co-workers accomplished a general and scalable total synthesis of halichondrins [59]. In this paper, a new Zr/Ni-mediated one-pot ketone synthesis gave the key to couple the right (**85**) and the left (**86**) halves of all types of halichondrins, including homo- and reluctant norhalichondrins (Scheme 15). Then, after fluoride deprotection of the silyl groups, ketone **87** was transformed to the spiroketal **88**. By coupling different halves, halichondrins, norhalichondrins and homohalichondrins were accessed. To exemplify the success of this strategy, an overall yield of 14.3% was obtained for the synthesis of halichondrin B from commercial d-galactal.

**Scheme 16:**
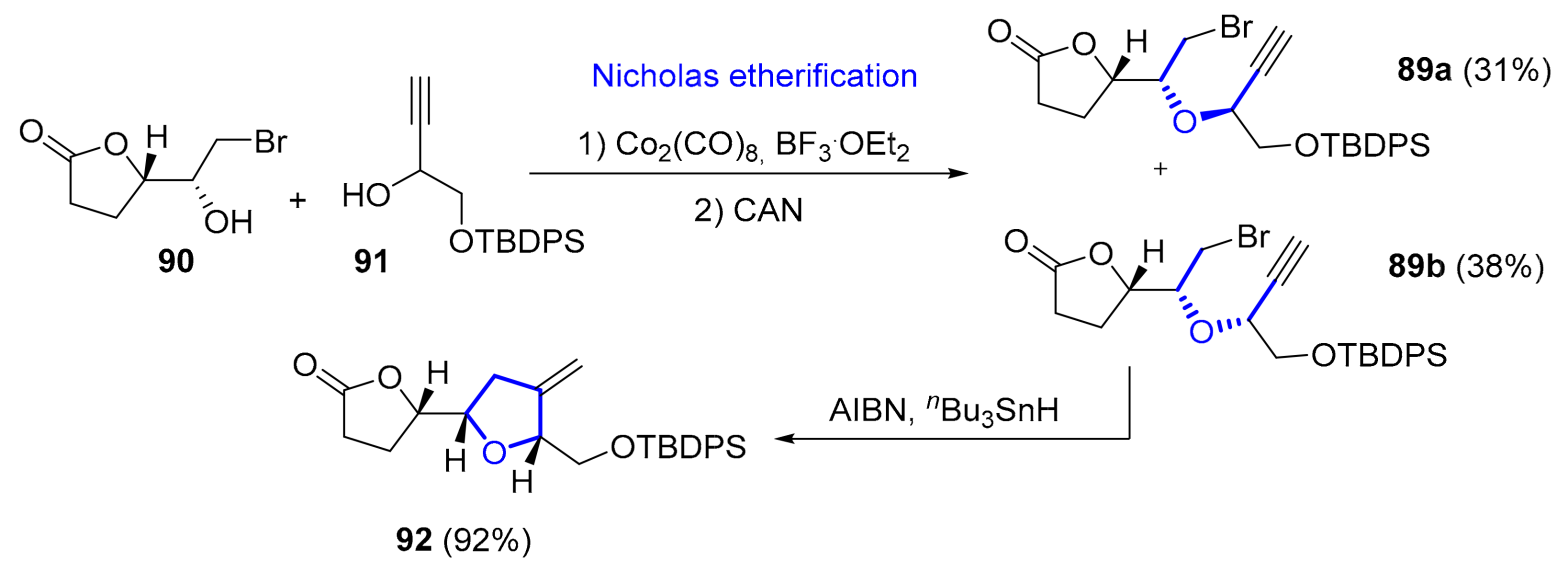
Reverse approach to halichondrin B by Nicolaou.

Recently, Nicolaou and coworkers described a reverse approach for the total synthesis of halichondrin B [60]. The strategy consisted of first forming the C-O bonds and then the C-C bonds of the cyclic moieties, which is opposite of the usual methods that first form C-C bonds and then rely on C-O bond-forming cyclizations. As an example, we can see in Scheme 16 the formation of linear ethers **89a** and **89b** by Nicholas etherification of alcohols **90** and **91**. The required diastereoisomer **89b** was subjected to radical cyclization to close the THF ring in **92**. This methodology was applied throughout the total synthesis of halichondrin B, which was synthesized in just 25 linear steps from commercial materials.

**Figure 13:**
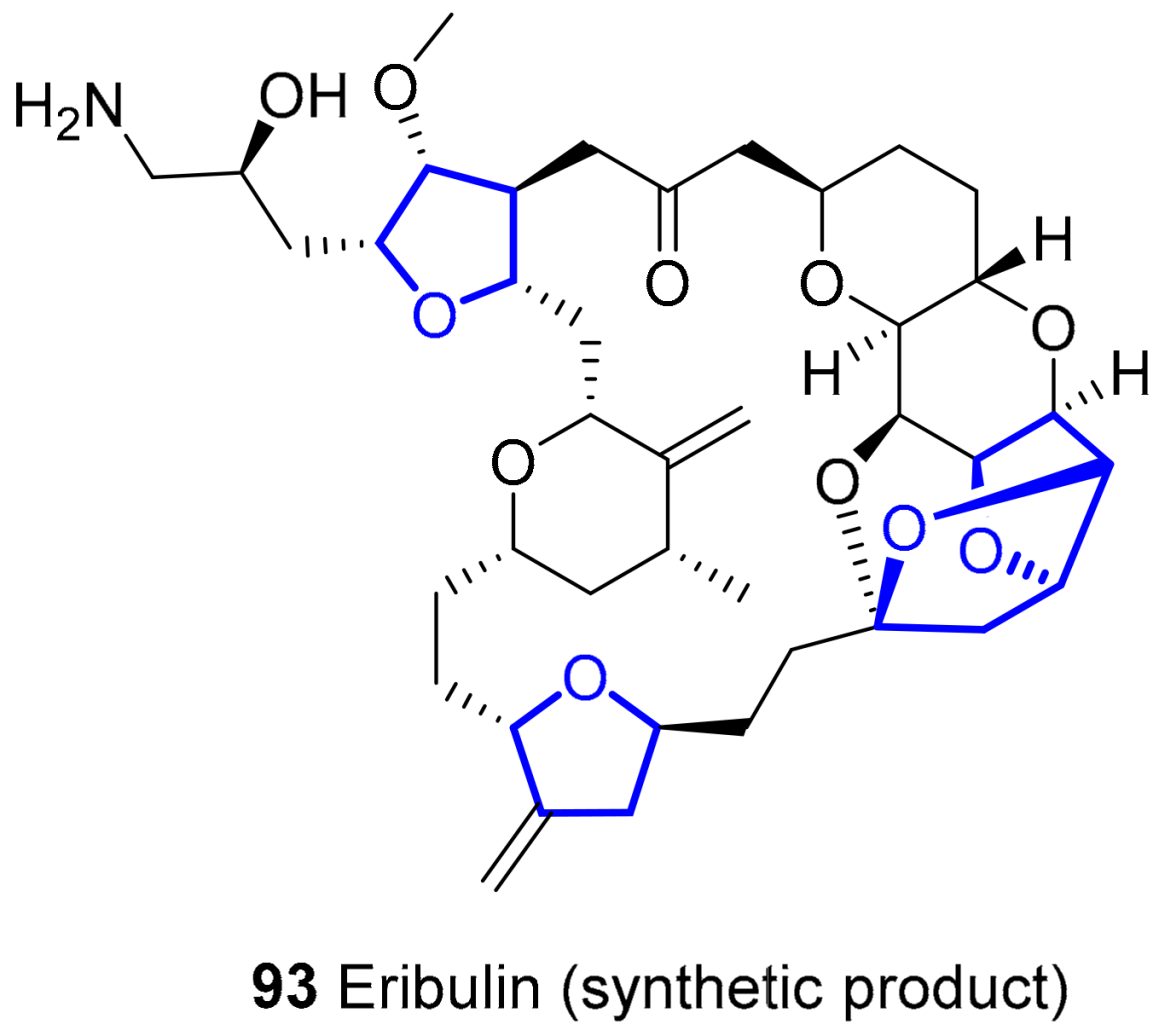
Structure of the anticancer drug eribulin.

Biological studies demonstrated that the right half of this class of natural products showed potent in vitro and in vivo antitumor activity [61], which led to the approval of eribulin mesylate (Figure 13) by the FDA for the treatment of late-stage breast cancer. This compound is known under the commercial name of Halaven^®^. The mechanism and pharmacokinetics of eribulin and its use in numerous Phase I, II, and III clinical trials have been described in different reviews [62,63].

#### 2.1.6. Iriomoteolides

**Figure 14:**
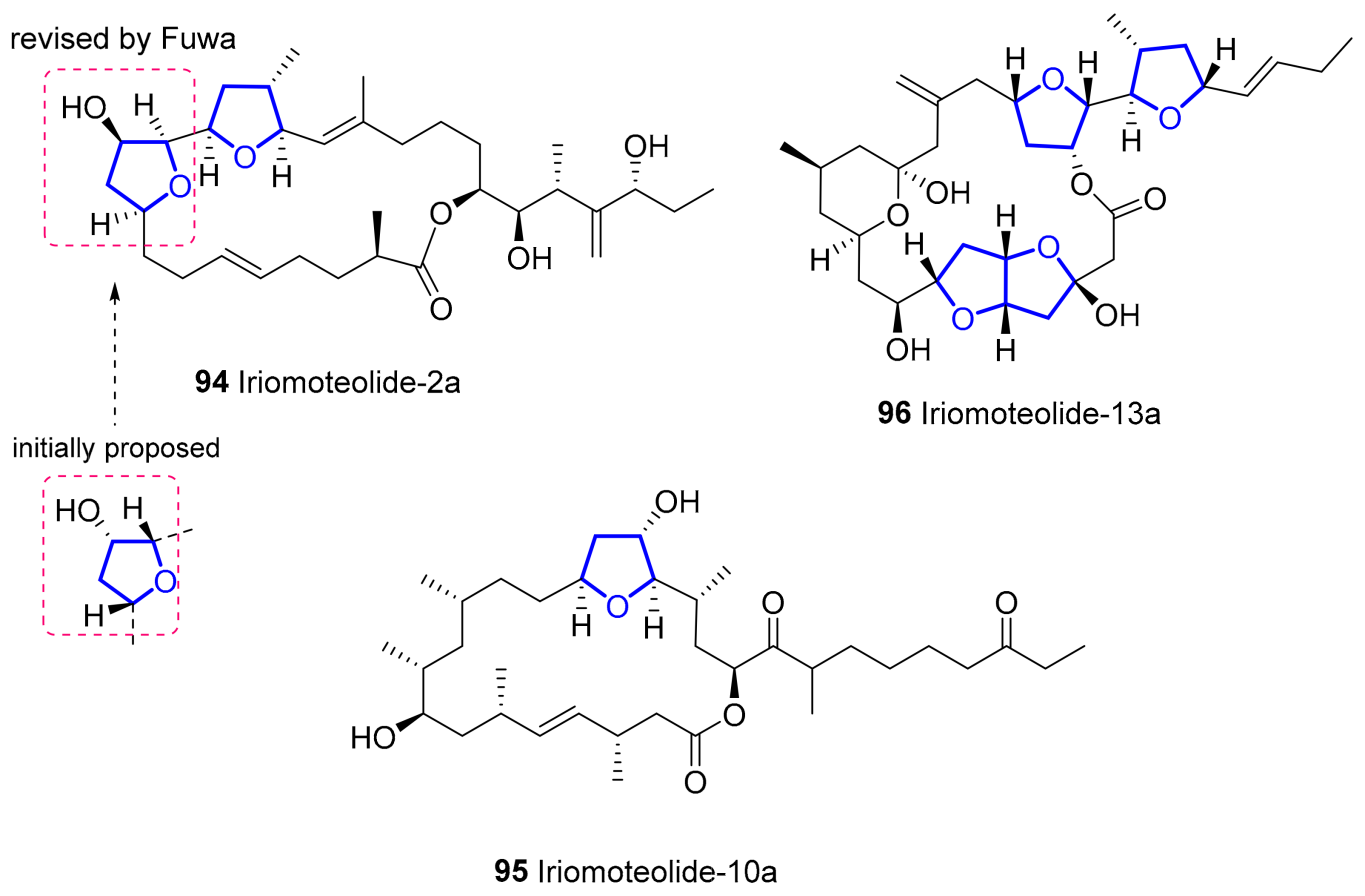
Structure of THF-containing iriomoteolides.

Iriomoteolides are a recent class of macrolides isolated from a marine benthic dinoflagellate of the *Amphidinium* species [64]. Among all members of this family, iriomoteolide-2a (**94**) [65], 10a (**95**) [66], and 13a (**96**) [67] present at least a substituted tetrahydrofuran ring (Figure 14). They stand up for their potent biological activity against human cervix adenocarcinoma HeLa cells (IC50 = 0.03 μg/mL, 1.5 μM and 0.5 μg/mL, respectively) and other cell lines, human B lymphocyte DG-75 (IC50 [2a] = 6 ng/mL and IC50 [10a] = 1.2 μM), and murine hepatocellular carcinoma MH134 cells (IC50 [10a] = 3.3 μM).

Despite their biological interest, there is no total synthesis reported for the 21-membered macrolide **95**, nor the 22-membered macrolide **96**, probably due to their complexity. On the contrary, the synthesis of **94** was accomplished by Fuwa and co-workers recently and it led to a structural revision [68,69].

**Scheme 17:**
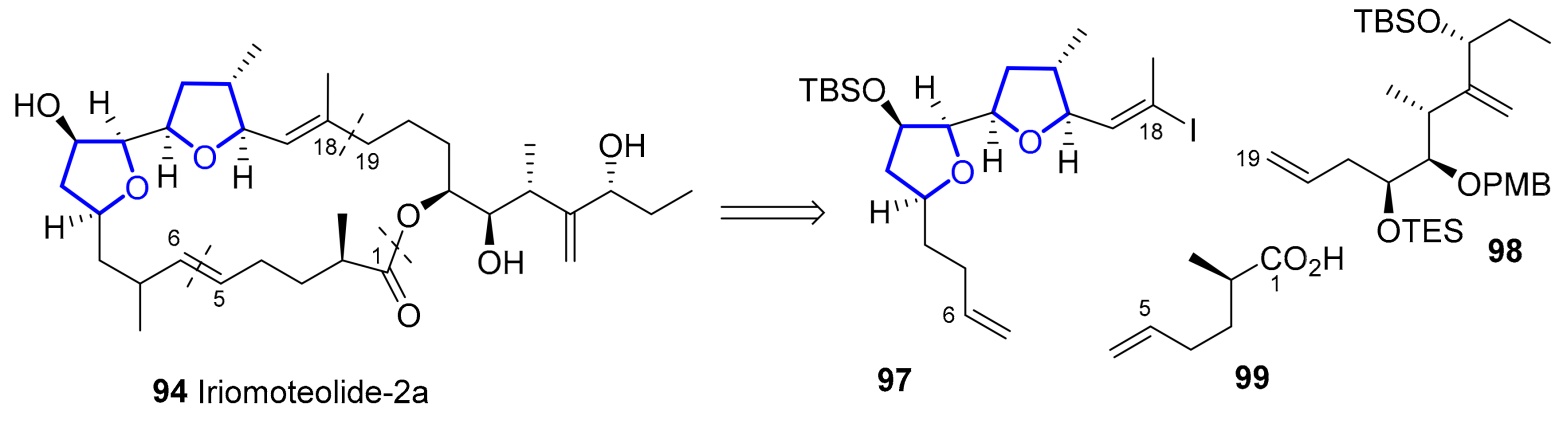
Retrosynthesis of iriomoteolide-2a.

Iriomoteolide-2a was the first 23-membered macrolide isolated from nature. Its structure contains two contiguous tetrahydrofuran rings embedded in the macrolide skeleton and a side chain containing three chiral centers. Two key steps made up the synthetic approach devised by Fuwa [68], a Suzuki-Miyaura coupling reaction between a vinyl iodide **97** and an olefin **98**, and a ring closing metathesis with **99** to build the macrolactone (Scheme 17).

**Scheme 18:**
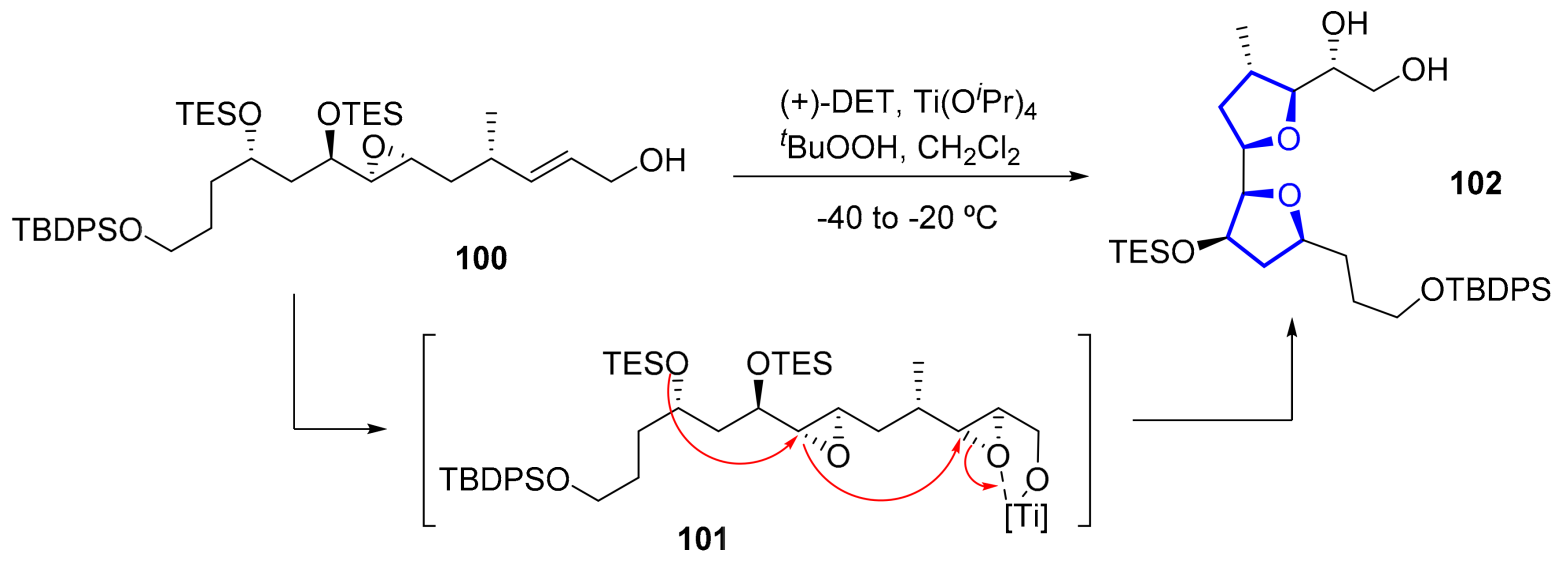
Synthesis of contiguous THF in iriomoteolide-2a.

A convergent strategy allowed the synthesis of the different possible stereoisomers of iriomoteolide-2a. Having discarded the original proposed configuration, the construction of the correct bis-THF fragment was achieved by two sequential cycloetherifications. Thus, Sharpless asymmetric epoxidation of **100** provided diepoxide **101**, which in situ underwent epoxide opening cascade with formation of the desired bis-THF **102**. The relative configuration of this bis-tetrahydrofuran was established by ROE experiments (Scheme 18).

**Scheme 19:**
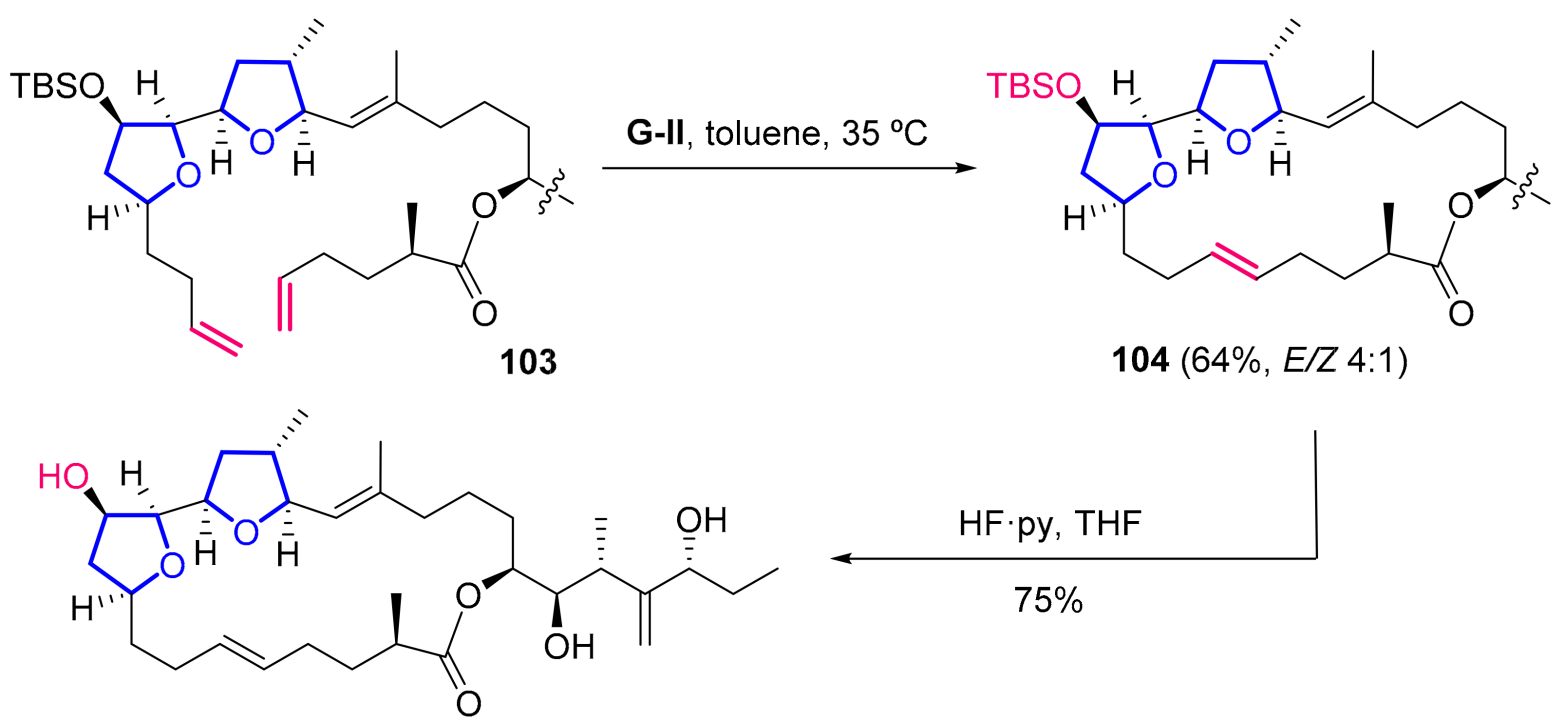
Engame to iriomoteolide-2a by Fuwa.

In this synthesis macrocyclization was performed at a very late stage. Thus, ring closing methathesis of precursor **103**, with a second-generation Grubbs catalyst (G-II), led to macrocycle **104**, which after final deprotection step afforded the desired iriomoteolide-2a (Scheme 19).

Thanks to this total synthesis it was possible to establish the absolute configuration of iriomoteolide-2a and to re-study the biological activity of synthetic compounds [69]. By comparing the spectral data, the correct stereochemistry of the natural compound could be assigned, which differed from the first proposed assignment [65]. Surprisingly, in contrast to the potent cytotoxic activity reported for the natural product, the synthetic iriomoteolide-2a only showed marginal antiproliferative activity in HeLa cells (IC50 = 60 μM). A plausible explanation for the high cytotoxicity measured in natural iriomoteolide-2a is the presence of traces of a highly potent contaminant in the sample. 

#### 2.1.7. Mandelalides

**Figure 15:**
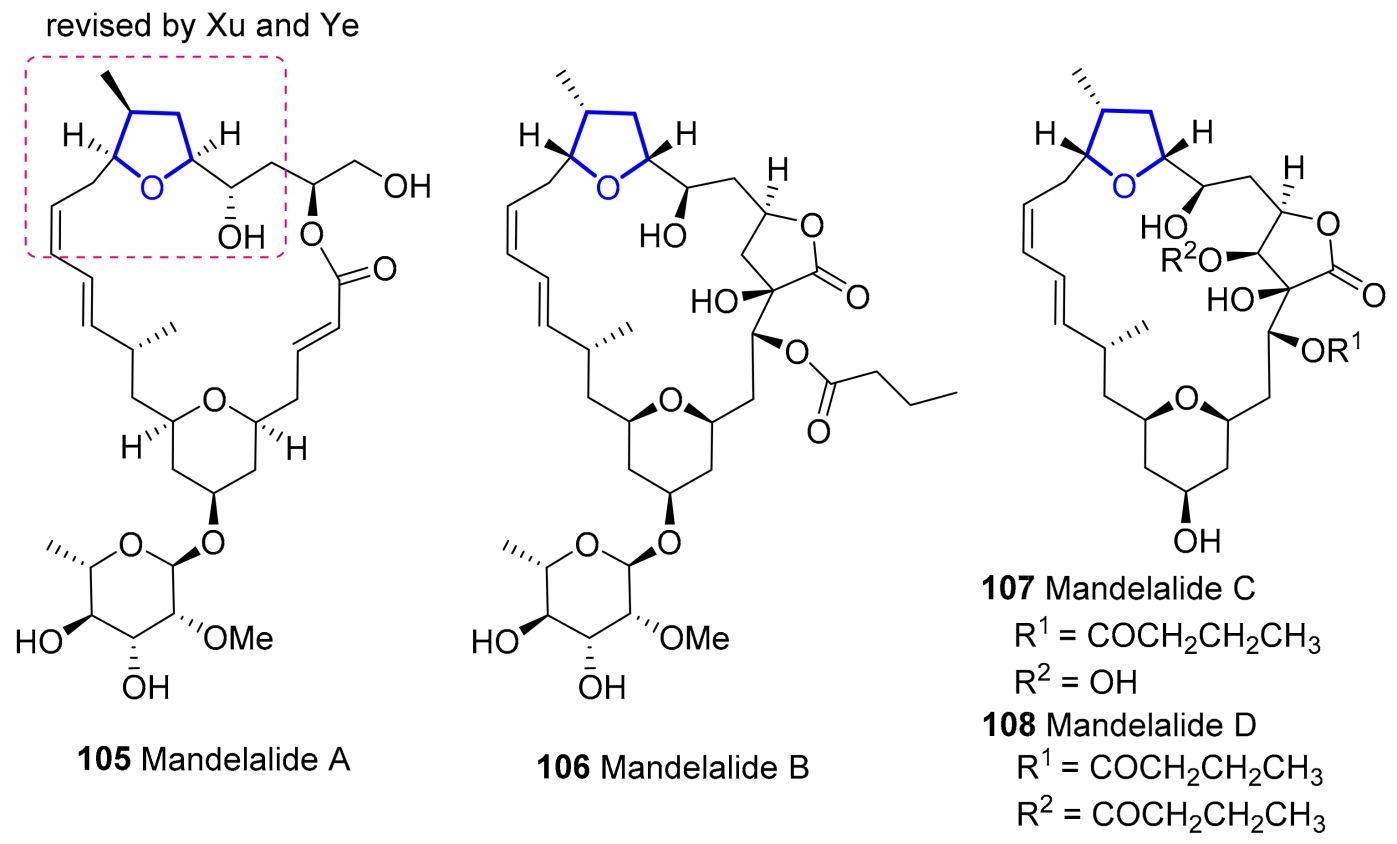
Structure of mandelalides.

Mandelalides A-D (**105**-**108**) (Figure 15) are a group of marine macrolides isolated in 2012 from a species of *lissoclinum* ascidian [70]. Their macrocyclic core has two cyclic moieties embedded, a glycosylated 2,6-*cis*-substituted tetrahydropyran and a 2,5-*cis*-substituted tetrahydrofuran. Within them, the natural isolated compounds **105** and **106** yielded nanomolar IC50 values against mouse Neuro2A neuroblastoma cells and human NCI-H460 lung cancer cell lines. Their complex structure was elucidated by 1D and 2D NMR experiments and mass spectrometry, although the stereochemistry of Mandelalide A was definitively established after its total synthesis by Xu and Ye [71].

Years later, new members of this family were isolated, namely mandelalide E-L [72,73]. This has permitted the study of structure-activity relationship, showing that glycosylation is essential for their biological activity.

Since the discovery of mandelalide A, different researchers have proposed approaches for its total synthesis [12]. The main difference between them lies in the key reaction steps. Tao Ye and co-workers reported the use of Rychnovsky–Bartlett cyclization for the preparation of the tetrahydrofuran moiety and an Horner–Wadsworth–Emmons for the macrocyclization [71]. Amos B. Smith developed an anion relay chemistry (ARC) strategy [74] to synthesize the tetrahydrofuran and tetrahydropyran structural motifs which were joined by Yamaguchi esterification [75]. Intramolecular Heck cyclization [76], Sharpless asymmetric dihydroxylation [77] or Julia olefination [78] were also employed in other total syntheses. 

**Scheme 20:**
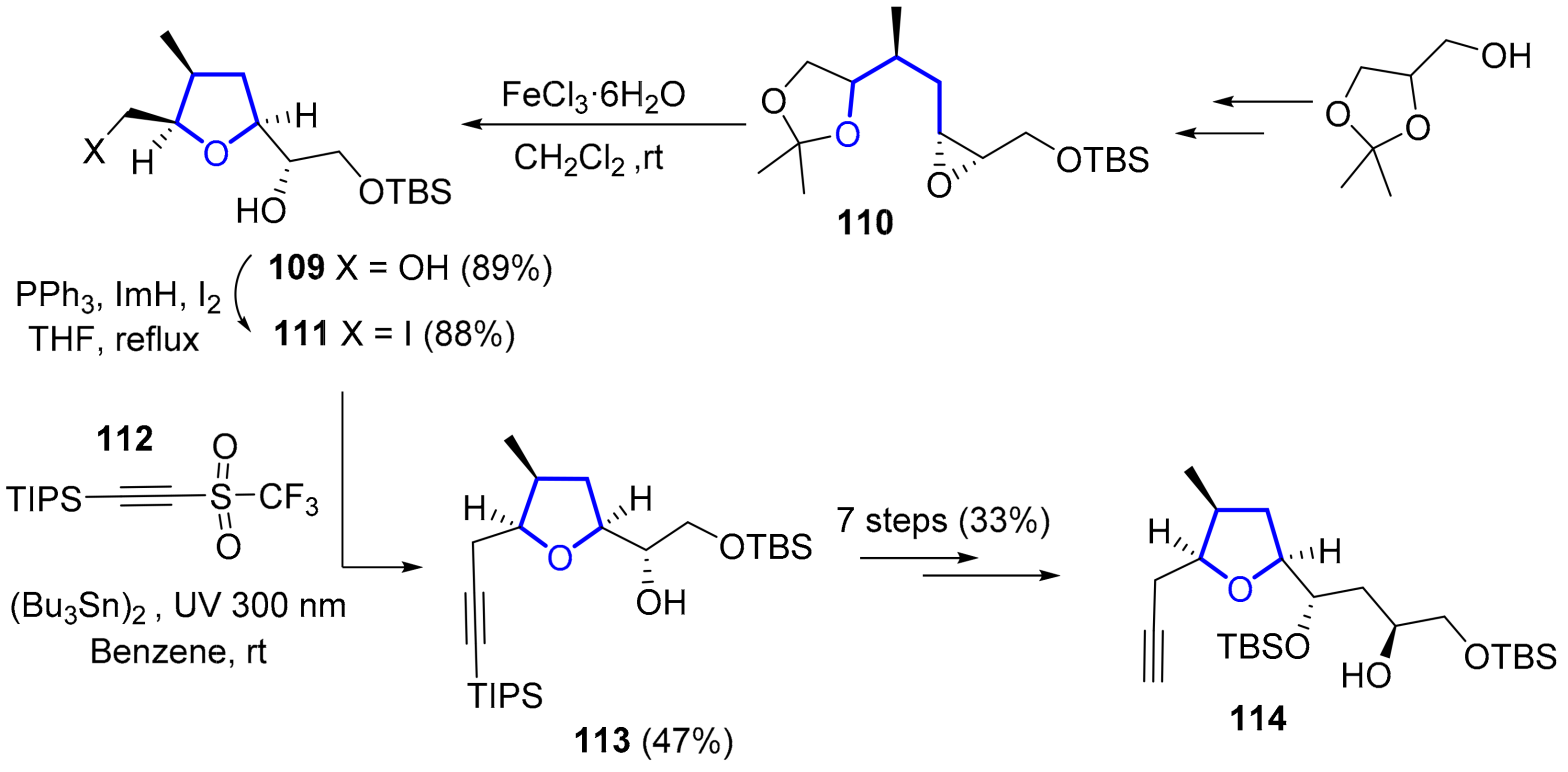
Synthesis of the trisubstituted THF in mandelalides.

As an example, we want to highlight the convergent total synthesis reported by Altmann [79]. In this synthesis, the highly oxygenated tetrahydrofuran **109** was constructed from compound **110** by acetal cleavage/epoxide opening cascade reaction. Then, substitution of the primary OH with iodine (**111**) and subsequent radical alkynylation with sulfone **112** afforded **113**. After further elaboration, the tetrahydrofuran fragment **114** was accessed (Scheme 20). 

**Scheme 21:**
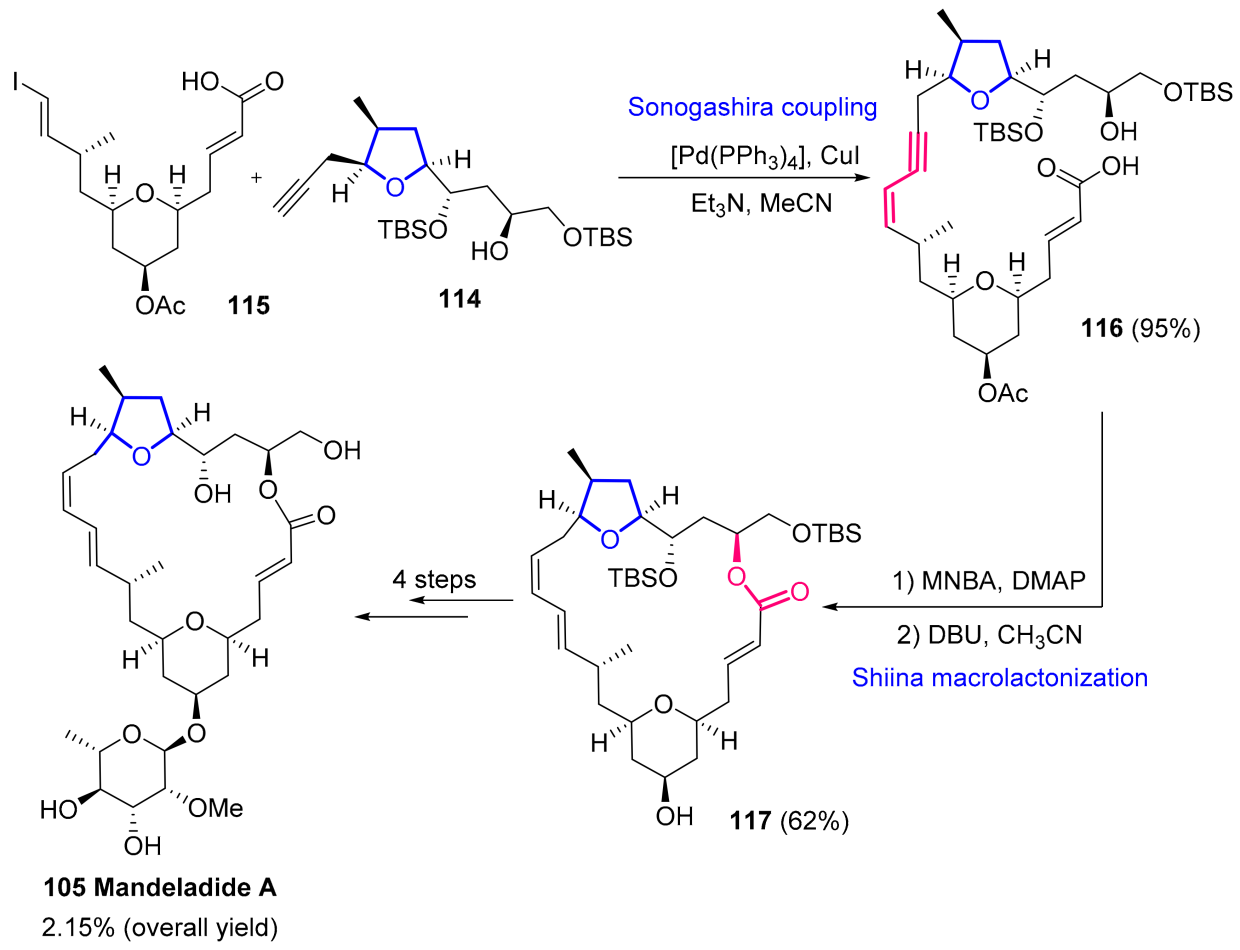
Macrocyclization and final steps for the synthesis of mandelalide A.

With the needed building blocks in hand, tetrahydropyran **115** and tetrahydrofuran **114** moieties were united via Sonogashira coupling to afford enyne **116**. Then, Shiina macrolactonization was used to access macrolactone **117**, which was transformed to the desired natural product in four additional steps and a 2.15% overall yield (Scheme 21). 

Biological studies of synthetic mandelalide A revealed a potent inhibitory activity of the proliferation of H460 and A549 lung carcinoma cells, but not cytotoxic activity at least within the concentration range studied [70,79]. In general, mandelalides have shown to have highly cell-type dependent bioactive effects.

#### 2.1.8. Mangromicins

**Figure 16:**
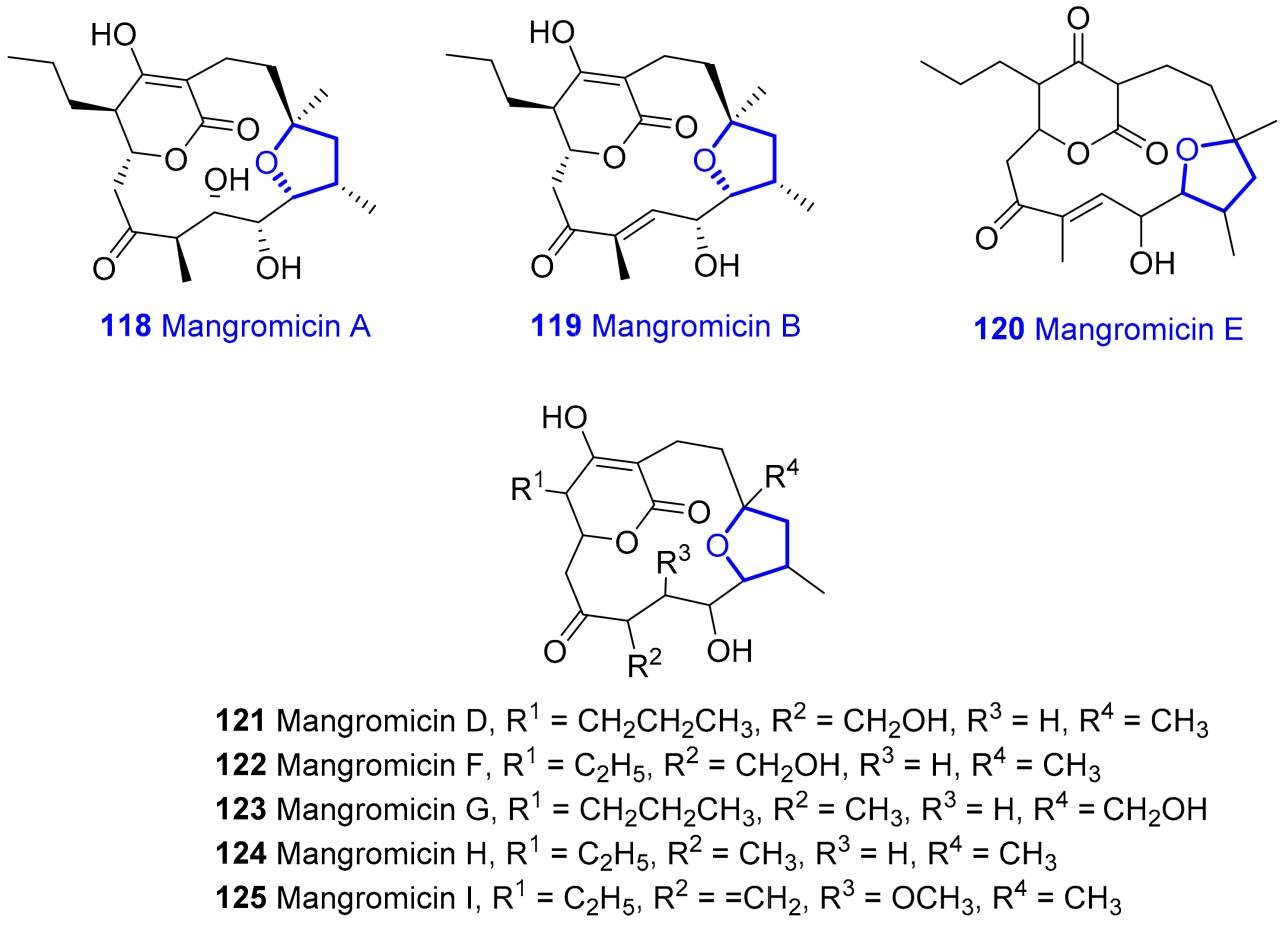
Structure of mangromicins.

Mangromicin A (**118**) and B (**119**) (Figure 16) were the first members of this family isolated from actinomycete *Lechevalieria aerocolonigenes* [80], followed by six new analogues, mangromicins D-I [81]. The mangromicins contain a cyclopentadecaene skeleton with a 5,6-dihydro-4-hidroxy-2-pyrone moiety and a tetrahydrofuran unit. All mangromicins show important biological activities. Special effects have been found in Mangromicin A, which exhibits potent antitrypanosomal activity against Trypanosoma brucei brucei GUTat 3.1 strain (IC50 = 2.4 in vitro essays) and cytotoxicity against MRC-5 cells. 

**Scheme 22:**
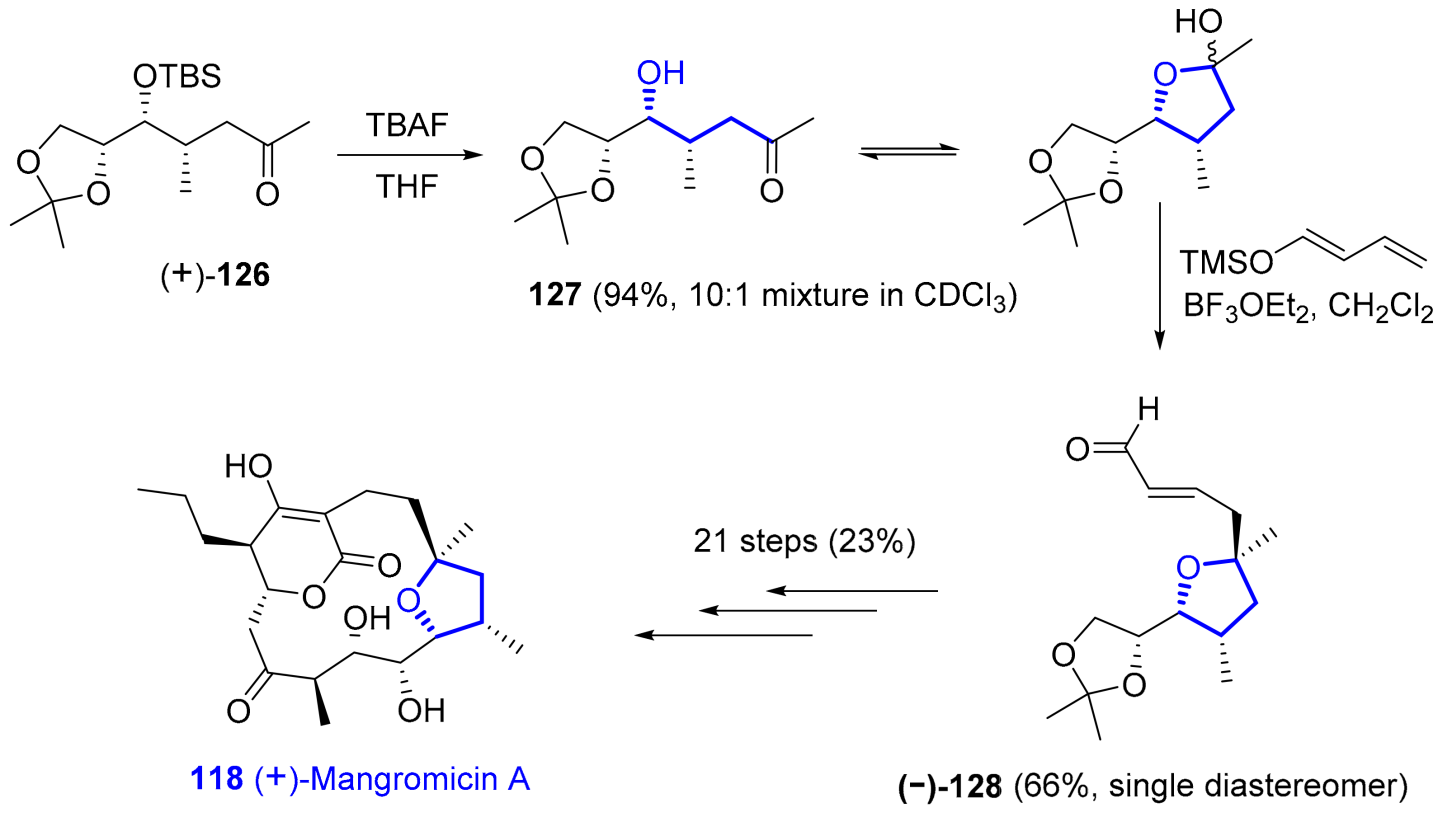
Synthesis of THF segment of mangromicin A.

The first and unique enantioselective total synthesis of mangromicin A was reported by Takahashi and coworkers [82]. Deprotection of the OTBS group in compound **126** led to hydroxyketone **127**, in equilibrium with its hemiketal. Then, a Mukaiyama-type vinylogous alkylation was used as key step to synthesize the desired tetrahydrofuran moiety (-)-**128**, bearing a C-2 quaternary carbon with the desired configuration. A further 21 steps, including a crucial Dieckmann cyclization to generate the 4-hydroxydihydropyrone unit, were needed to complete the total synthesis of mangromicin A (Scheme 22) [82].

#### 2.1.9. Nonalides: Cytospolides

**Figure 17:**
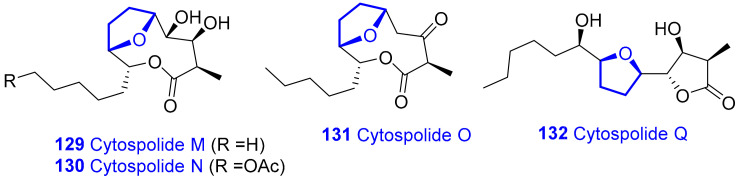
Structure of cytospolides.

Cytospolides (Figure 17), which belong to a nonalide family, are a group of compounds which were isolated in Gomera island (Spain) from an endophytic fungus, *Cytospora* sp., by Zhang and co-workers in 2011 [83]. The different structures and absolute configurations of cytospolides were first elucidated and established by spectroscopic analysis, chemical derivatization, and X-ray diffraction [83]. Almost all members of this family contain a 10-membered lactone. Additionally, cytospolides M (**129**), cytospolide N (**130**), and cytospolide O (**131**) are tetrahydrofuran-containing nonalides. Cytospolide Q (**132**) is the exception, containing a 15-carbon skeleton with two different rings, a tetrahydrofuran and a γ-butyrolactone.

**Scheme 23:**
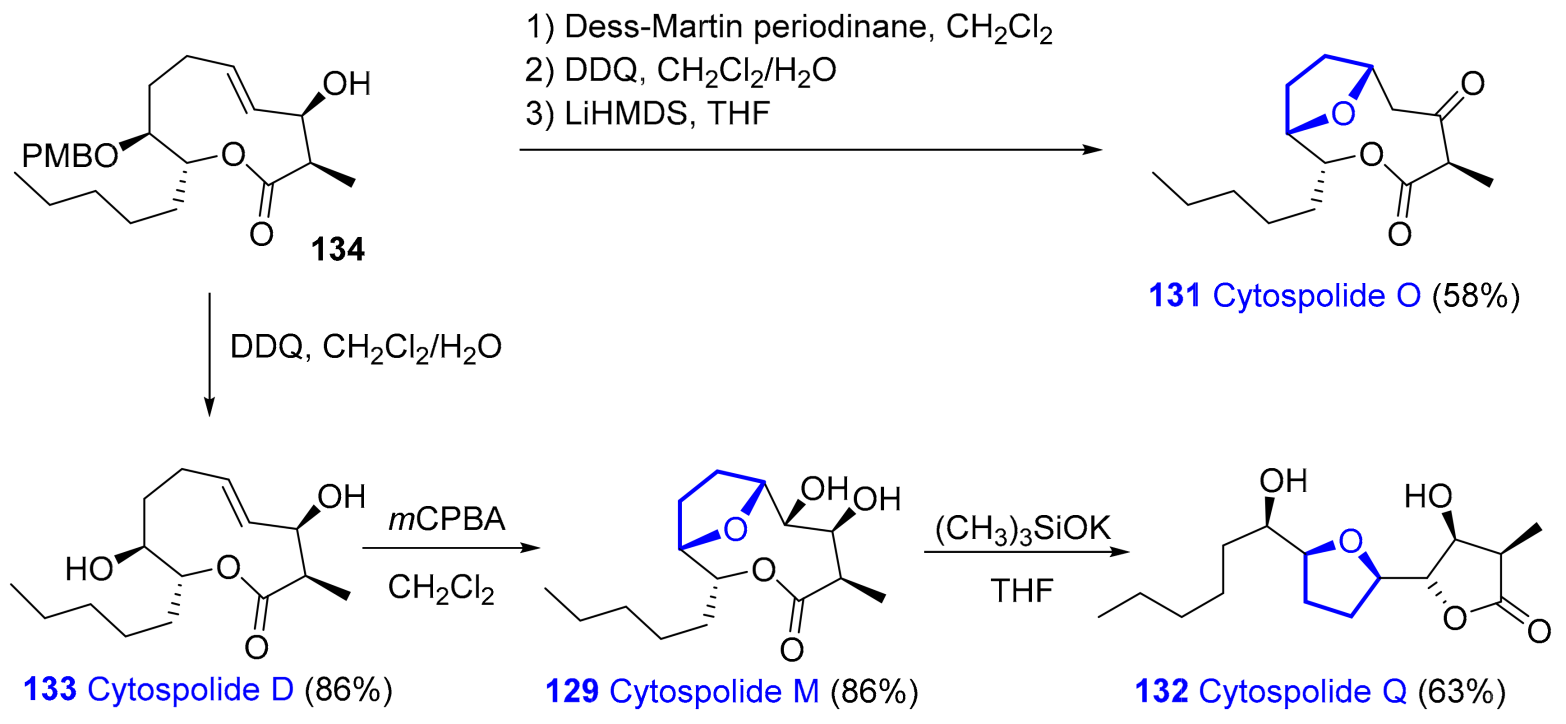
Synthesis of cytospolides.

Stark et al. have reported the total synthesis of cytospolide D and its conversion into cytospolides M, O, and Q (Scheme 23) [84]. Cytospolide M was obtained in a single step with a 86% yield from cytospolide D, by diastereoselective epoxidation. Subsequent opening of cytospolide M with potassium trimethylsilanolate, followed by spontaneous recyclization during the workup, allowed the preparation of the desired cytospolide Q. On the other hand, cytospolide O was obtained through an oxa-Michael addition from a close precursor of cytospolide D in three steps.

**Scheme 24:**
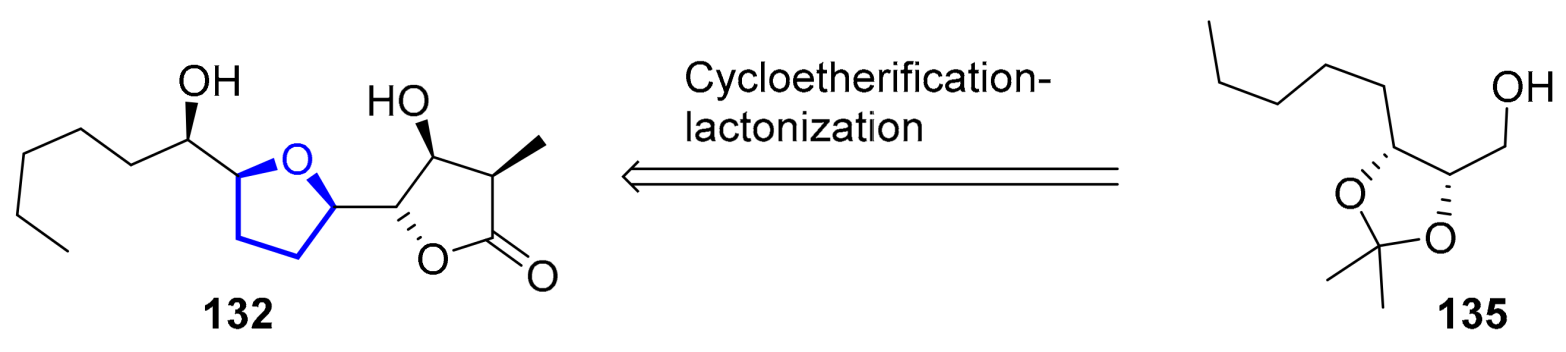
Retrosynthesis of cytospolide Q.

In a different approach, a convergent route for the total synthesis of cytospolide Q has been proposed in 10 linear steps, with an overall yield of 2.8%, from known intermediate 135 [85]. A set of cascade reactions are the key to build the tetrahydrofuran ring and the γ-butyrolactone moiety (Scheme 24).

**Scheme 25:**
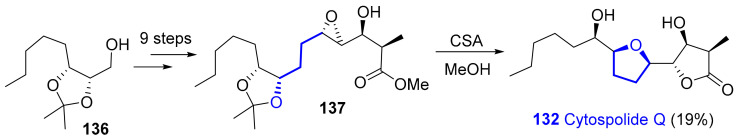
Total synthesis of cytospolide Q.

Nine linear steps from **136** were needed to prepare the required precursor (**137**). A set of convenient cascade reactions, such as acid-catalyzed acetal deprotection, subsequent tetrahydropyranyl formation by epoxide opening with the appropriate hydroxyl group and final γ-lactonization allowed the formation of cytospolide Q in a single step from **137** (Scheme 25).

**Scheme 26:**
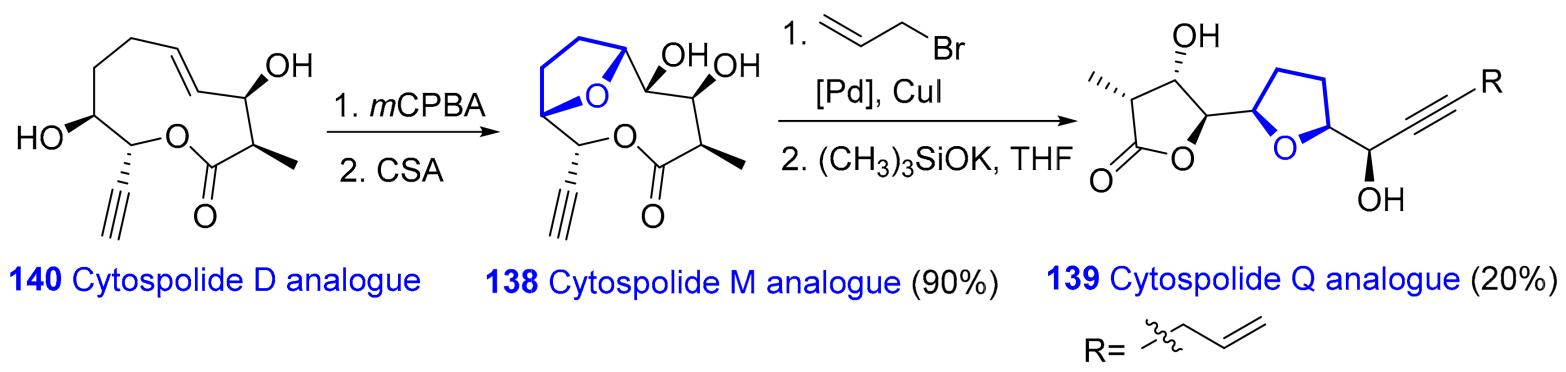
Synthesis of cytospolide analogues.

The moderate cytotoxic activity against tumor cell lines of some members of the cytospolide family [83] has prompted researchers to synthesize structurally diverse derivatives which may have improved properties [85]. Thus, Stark and Erlich [86] envisioned the synthesis of cytospolide analogues **138** and **139** from a modified cytospolide D intermediate **140** in which an alkynyl side chain was used as a versatile handle for further functionalization (Scheme 26). 

#### 2.1.10. Oscillariolide

**Figure 18:**
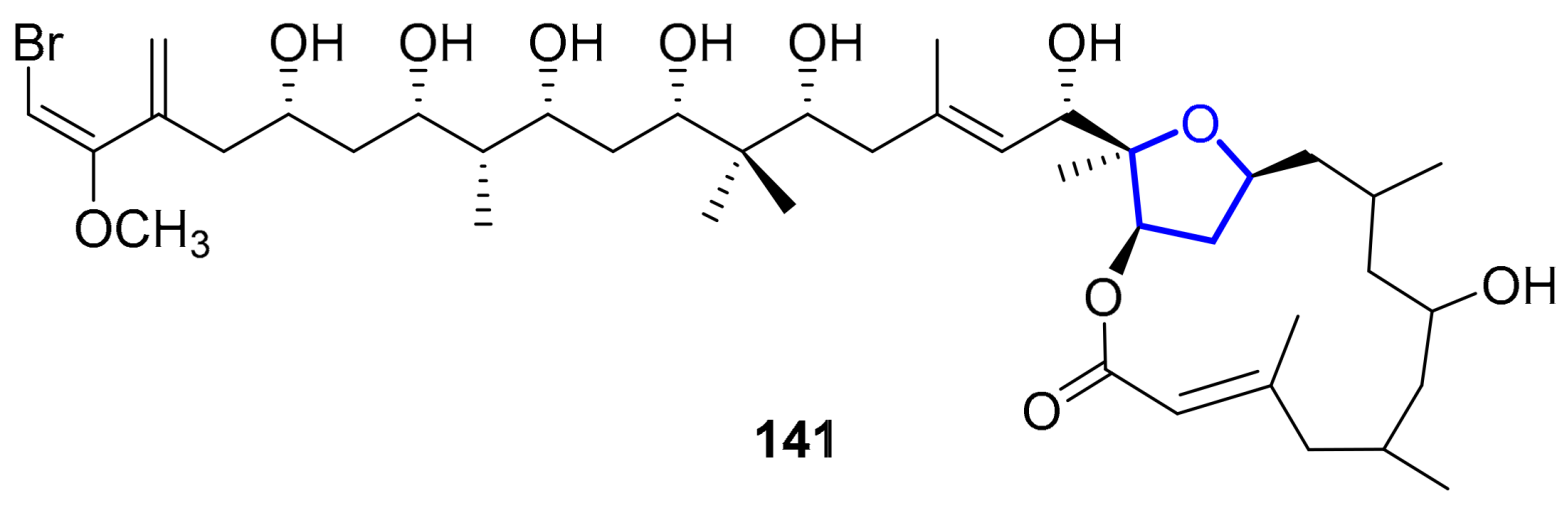
Structure of oscillariolide.

Murakami and co-workers isolated a new macrolide from a marine blue-green alga *Oscillatoria* sp. in 1991 [87]. Oscillariolide (Figure 18) has a complex structure which was established on the basis of extensive spectral analysis. Interestingly, its determination has been very useful for the discovery of phormidolides.

Despite the interesting inhibition activity of oscillariolide towards the cell division of fertilized starfish eggs, no total synthesis of this compound has been reported.

#### 2.1.11. Phormidolides

**Figure 19:**
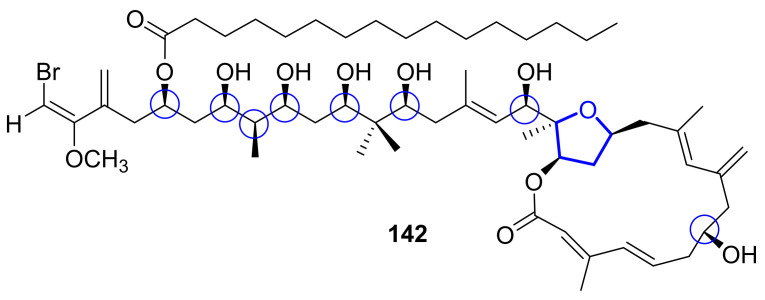
Structure of phormidolide A.

The first member of this family, Phormidolide A (**142**) (Figure 19), was isolated in 2002 from the marine cyanobacterium *Leptolyngbya* sp. [88]. Although it was an inactive metabolite in cell line essays, phormidolide A showed high toxicity to brine shrimp (LC50 = 1.5 μM). At that moment, a complex structure was proposed on the basis of spectroscopic techniques. Eleven stereocentres, a tetrahydrofuran-embedded macrolactone, a polyol side chain and a terminal bromomethoxydiene motif composed the polyketide. Almost 20 years later, the assignment of eight chiral centers was corrected thanks to the contribution of Gerwick, Paterson, Britton, and Piel and colleagues, who used synthetic methods [89], computational information, and anisotropic NMR studies [90].

**Figure 20:**
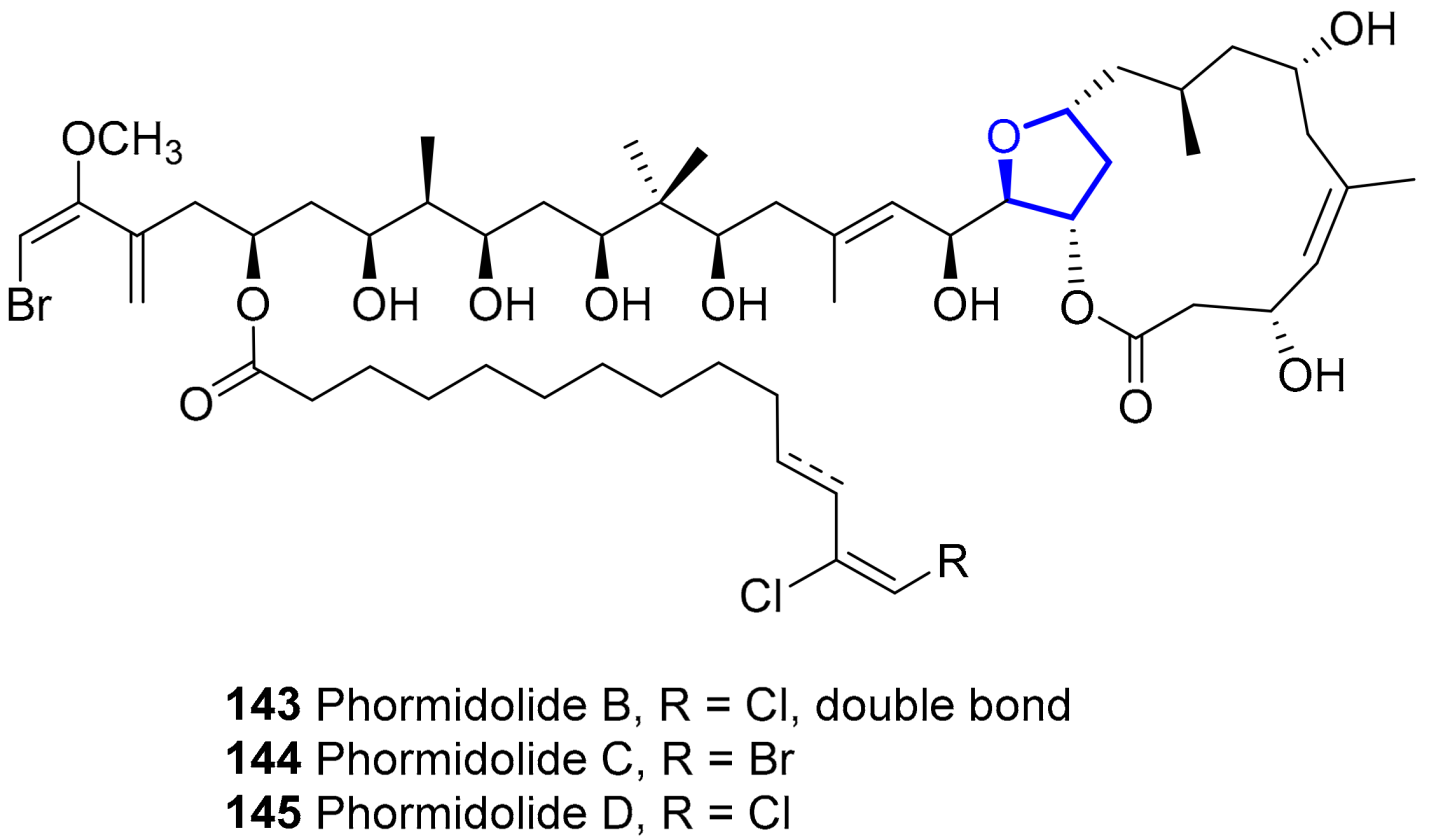
Structure of phormidolides B-D.

In 2015, the study of an active organic extract of *Petrosiidae* sponge concluded with the discovery of two important novel phormidolides, phormidolide B and phormidolide C (Figure 20) [91]. Their structural similarity to phormidolide A [90] and oscillariolide [87] helped in the establishment of their overall structure by NMR techniques. 

These new compounds present cytotoxic activity against three human tumor cell lines, lung (A-549), colon (HT-29), and breast (MDA-MB-231), with an unknown mechanism of action. Their challenging structure has attracted the interest of synthetic chemists who have developed different strategies to access either the macrolide ring [91,92,93] or the polyhydroxylated chain [94,95]. 

**Scheme 27:**
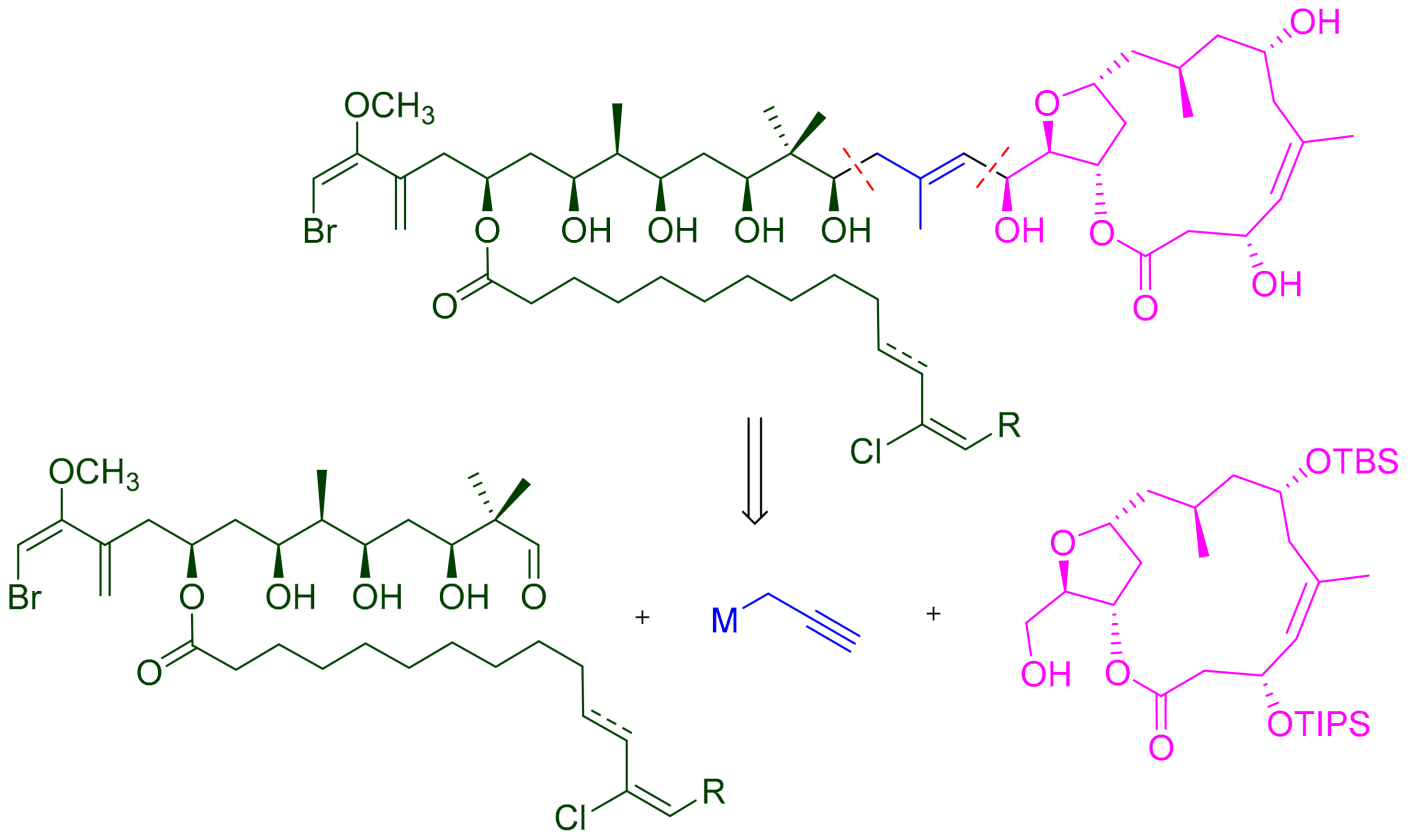
Retrosynthesis of phormidolides.

Phormidolides could be divided into three molecular fragments with two main disconnections: a macrocyclic core, a polyhydroxylated chain containing a tetradecanoic fatty acid, and a propargylic organometallic (Scheme 27). Synthetic approaches to each individual fragment have been described, although no completed synthesis of any of these compounds has been reported so far [93]. 

**Scheme 28:**
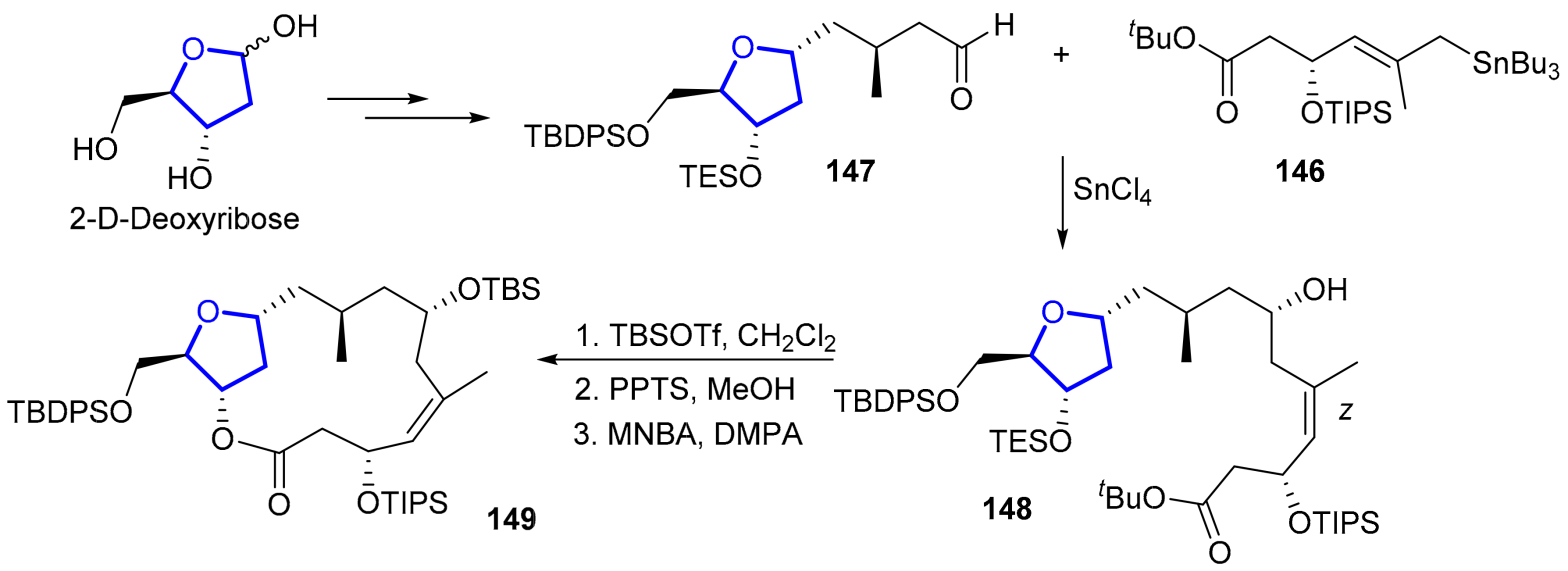
Retrosynthesis of phormidolide macrocycle.

Focusing on the tetrahydrofuran-containing macrolactone, Álvarez et al. published an approach to its synthesis starting from commercially available 2-D-deoxyribose [91]. The key steps of this synthesis are the simultaneous formation of a trisubstituted double bond and a new stereocenter through the stereoselective 1,5-*anti*-addition of an allylstannane **146** and ribose-derived aldehyde **147**. Then, **148** was subjected to final Shiina macrolactonization affording the desired **149** (Scheme 28). 

### 2.2. Linear Polyketides

#### Ionostatin

**Figure 21:**
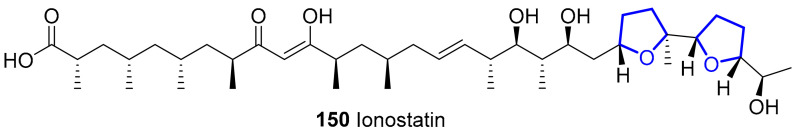
Structure of ionostatin.

**Figure 22:**
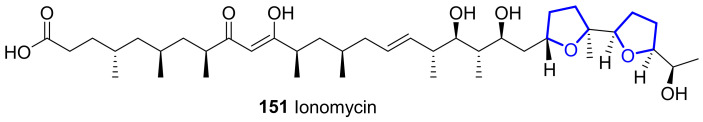
Structure of ionomycin.

Ionostatin (Figure 21) [96], extracted from an actinomycite of *Streptomycetaceae* family, is the most recently discovered polyether ionophore [97,98,99]. Its structure and absolute stereochemistry were determined by NMR experiments, X-ray diffraction (calcium salt) and bioinformatic approach. The compound, a close analog to ionomycin (Figure 22) [100], contains 15 chiral centers and two tetrahydrofuran rings.

Polyether ionophores are chemotherapeutic agents for the treatment of cancer [101]. Initial bioactivity essays of ionostatin revealed inhibition (LD50 = 7.4 μg/mL) against two important cancer cell lines such as U87 glioblastama and SKOV3 ovarian carcinoma.

### 2.3. Polycyclic Polyketides

#### Akaeolide

**Figure 23:**
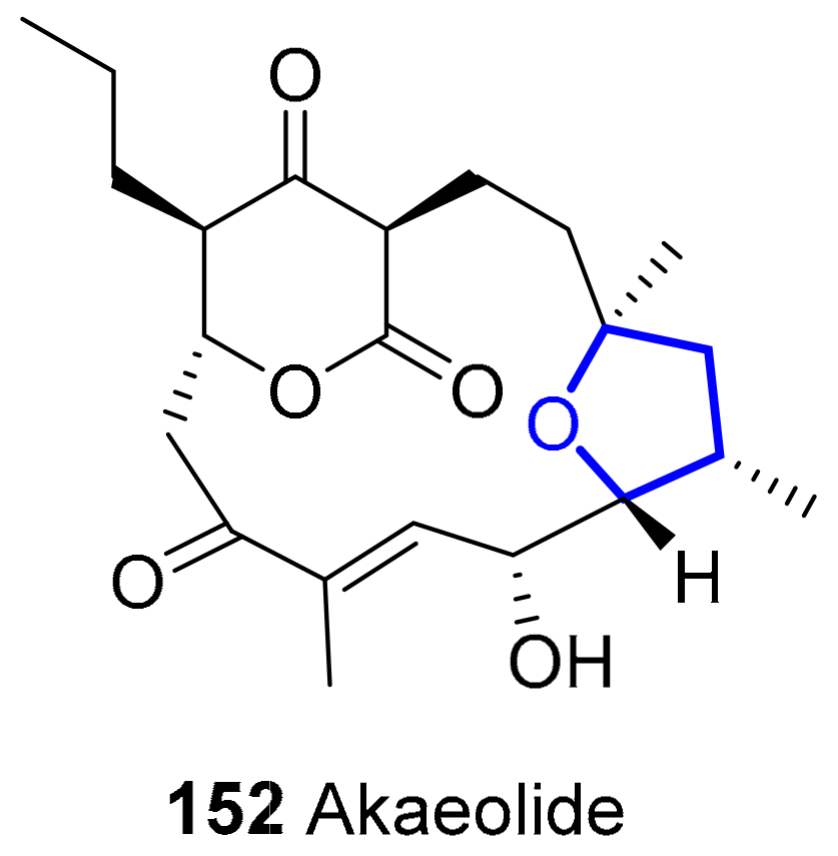
Structure of akaeolide.

Akaeolide (Figure 23) is another polycyclic polyketide isolated from a culture extract of a marine-derived actinomycete, *Streptomyces* sp., in 2013 [102]. Its 15-membered carbocyclic structure possesses a five-membered cyclic ether and a β-keto-δ-lactone unit. The biological essays of this compound have shown modest cytotoxicity against 3Y1 rat fibroblasts with an IC50 of 8.5 μM [103].

### 2.4. Acetogenin Metabolites

Acetogenins are secondary metabolites derived from polyketides. Acetogenins from marine algae are mostly halogenated and are thought to have a common C15 precursor derived from a C16 fatty acid. The majority of these marine C15 acetogenins are different-sized cyclic ethers with a terminal enyne or bromoallene. Among them, tetrahydrofuran and bis-tetrahydrofuran acetogenins are quite abundant. Acetogenins are known to be chemotaxonomic markers from red algae to the genus *Laurencia*. A general overview of C15 acetogenins from 1965 till 2015 was provided by Falkenberg [104].

In 2016, three new tetrahydrofuran-containing acetogenins with potent and selective antiproliferative activity against human nasopharyngeal carcinoma (NPC) cell lines and their methotrexate-resistant counterparts have been described [105].

**Scheme 29:**
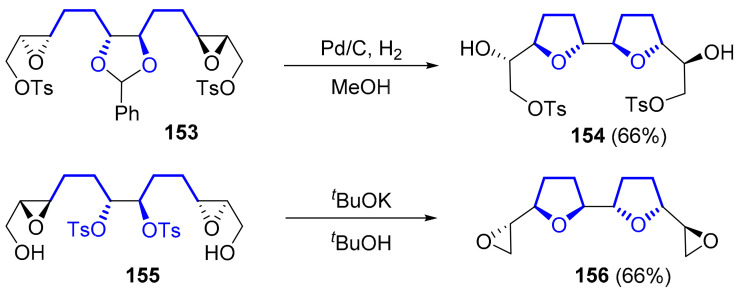
Synthesis of bis-THF core of acetogenins.

In 2019, a synthetic route for the bis-tetrahydrofuran core of acetogenins based on a chemoenzymatic cascade reaction was reported [106]. Catalytic hydrogenation of benzylidene acetal **153** produces the inside-out cyclization to afford bis-tetrahydrofuran-ditosylate **154** in 66% yield. It was also possible to obtain a different stereochemistry on the bis-tetrahydrofuran moiety **156**, through a double Payne rearrangement of epoxide **155** followed by 5-*exo*-tet cycloetherification (Scheme 29).

#### Obtusallenes

**Figure 24:**
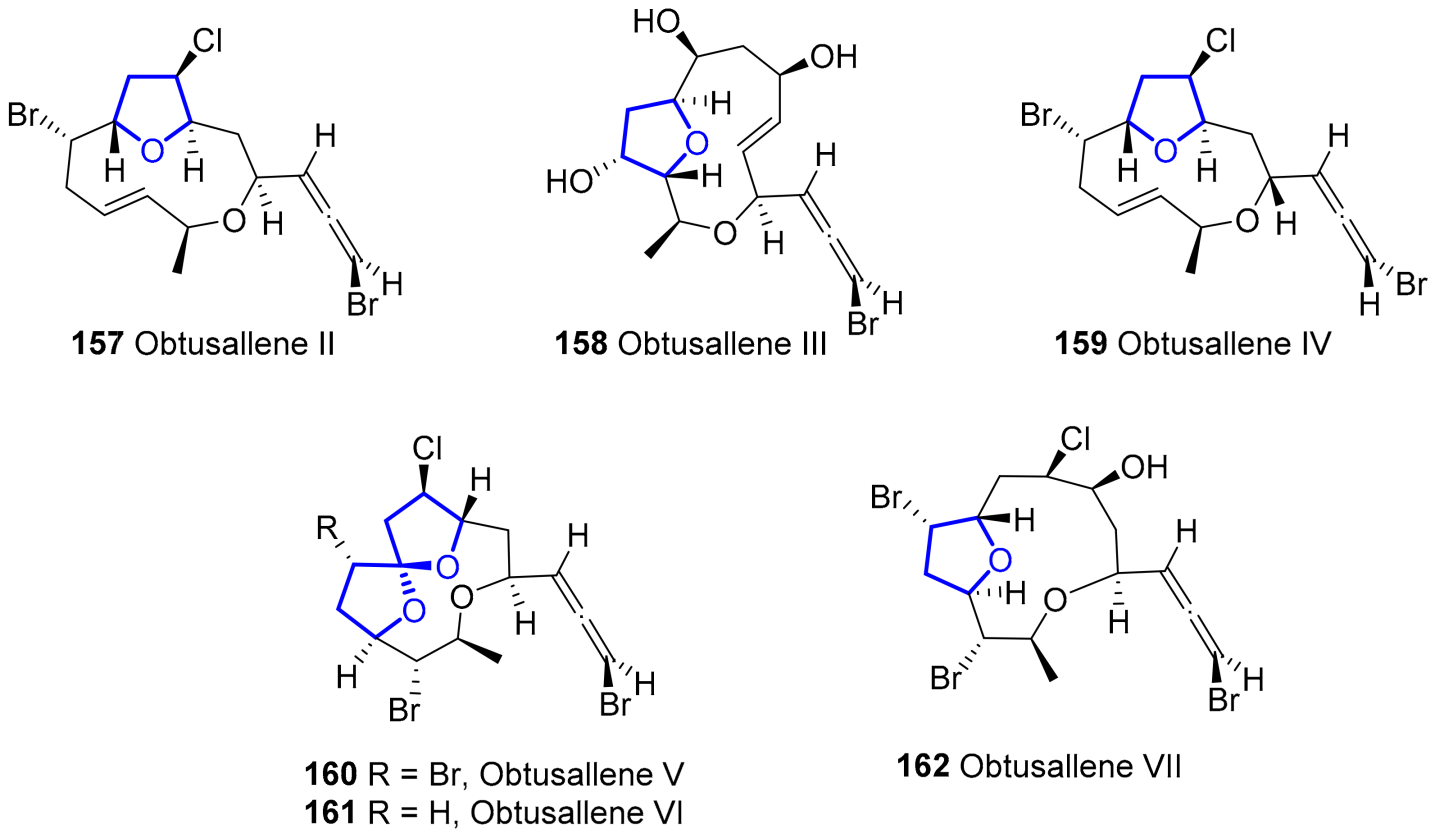
Structures of tetrahydrofuran-containing obtusallenes II-VII.

Obtusallenes are C15-halogenated acetogenins that contain a 12-membered ether ring. Obtusallene I was isolated in 1982 from red algae *Laurencia obtusa* and its structure was elucidated by NMR and X-ray crystallographic methods. Since, other obtusallenes have been described: obtusallene II (**157**) and III (**158**) [107], obtusallene IV (**159**) [108], obtusallenes V-VII (**160**-**162**), and obtusallenes VIII-IX [109]. The structure of **160**-**162** was corrected in 2008 by Braddock due to unambiguously solved X-ray crystallography and biosynthetic studies [110,111]. Among the obtusallenes, compounds **157**-**162** contain a tetrahydrofuran motif (Figure 24).

**Scheme 30:**
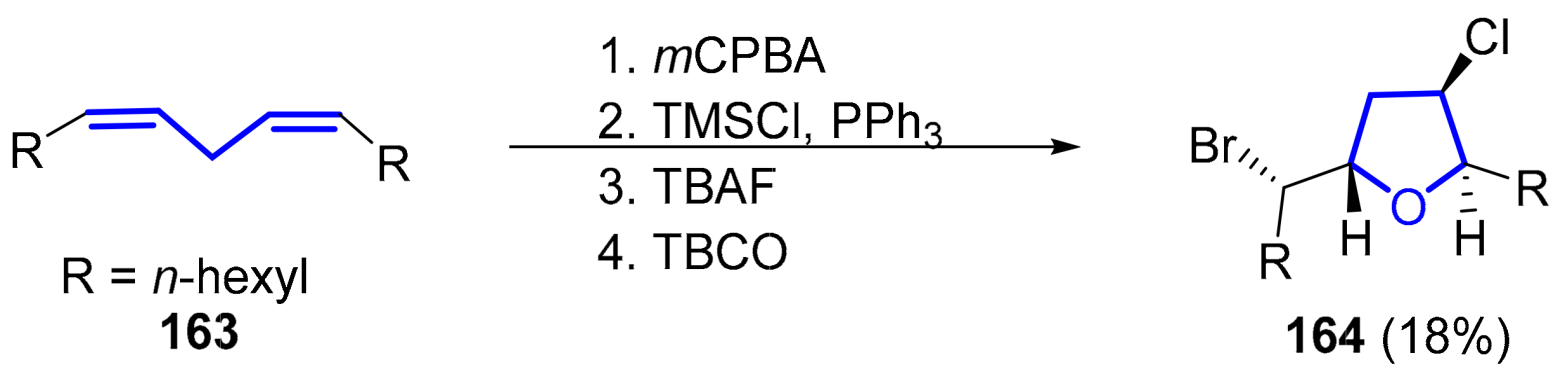
One pot reaction for THF core of obtusallenes 157 and 159.

The first synthesis of tetrahydrofuran rings of compounds **157** and **159** was reported in 2007 [112]. In this synthesis, diene **163** was transformed into tetrahydrofuran **164** with 18% yield and >95% purity in a one-pot reaction (with the sequential addition of *m*-CPBA, TMSCl and PPh_3_, TBAF, and then TBCO) (Scheme 30).

**Scheme 31:**
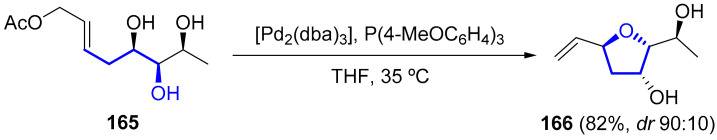
Synthesis of C8-C15 fragment of obtusallene **158**.

Parallelly, a synthesis of the C8–C15 fragment of obtusallene III **158** was described [113]. The Pd-catalyzed cyclization of triol **165** produced 2,5-disubstituted 3-hydroxytetrahydrofuran **166** with an 82% yield. The chemo- and diastereoselective cyclization implies the direct effect of a noncovalent interaction of the counterion carboxylate with the OH groups of the cationic π-allyl-Pd(II) intermediate (Scheme 31).

Braddock reported the only known total synthesis of a member of this family to date: obtusallene X (**167**) [114]. The key step of this synthesis is the cyclization of clorhydrine intermediate **168** through a stereoselective bromoetherification process.

**Scheme 32:**
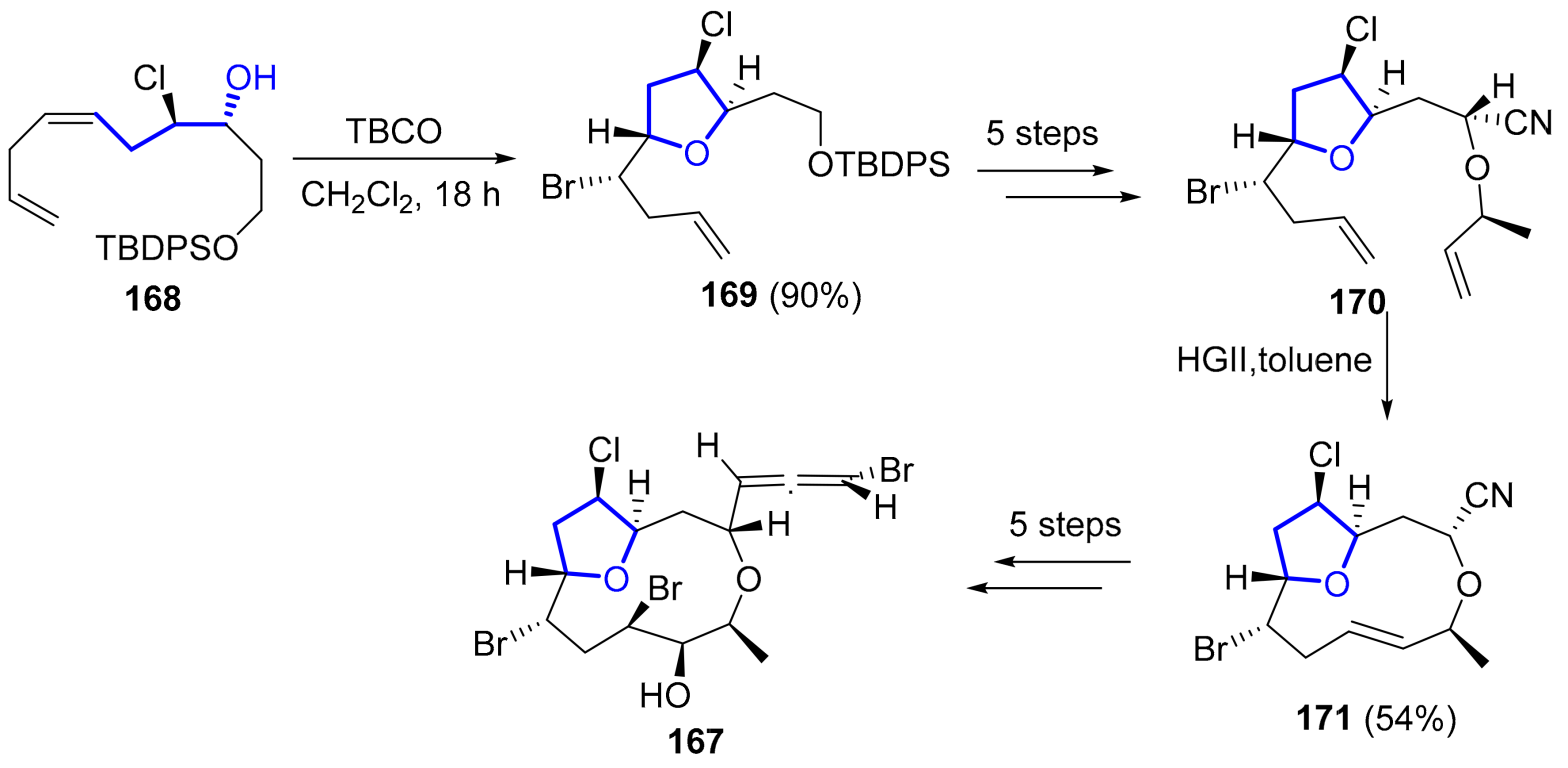
Synthesis of obtusallene X (**167**).

Five additional steps from **169** afforded acyclic diene **170**, which, under ring-closing metathesis with a second-generation Hoveyda−Grubbs precatalyst, produces macrocyclic **171**. Six additional steps were needed to obtain **167** with a 7% overall yield from **168** (Scheme 32). 

### 2.5. Polyhydroxyl

#### Amphezonol A

**Figure 25:**
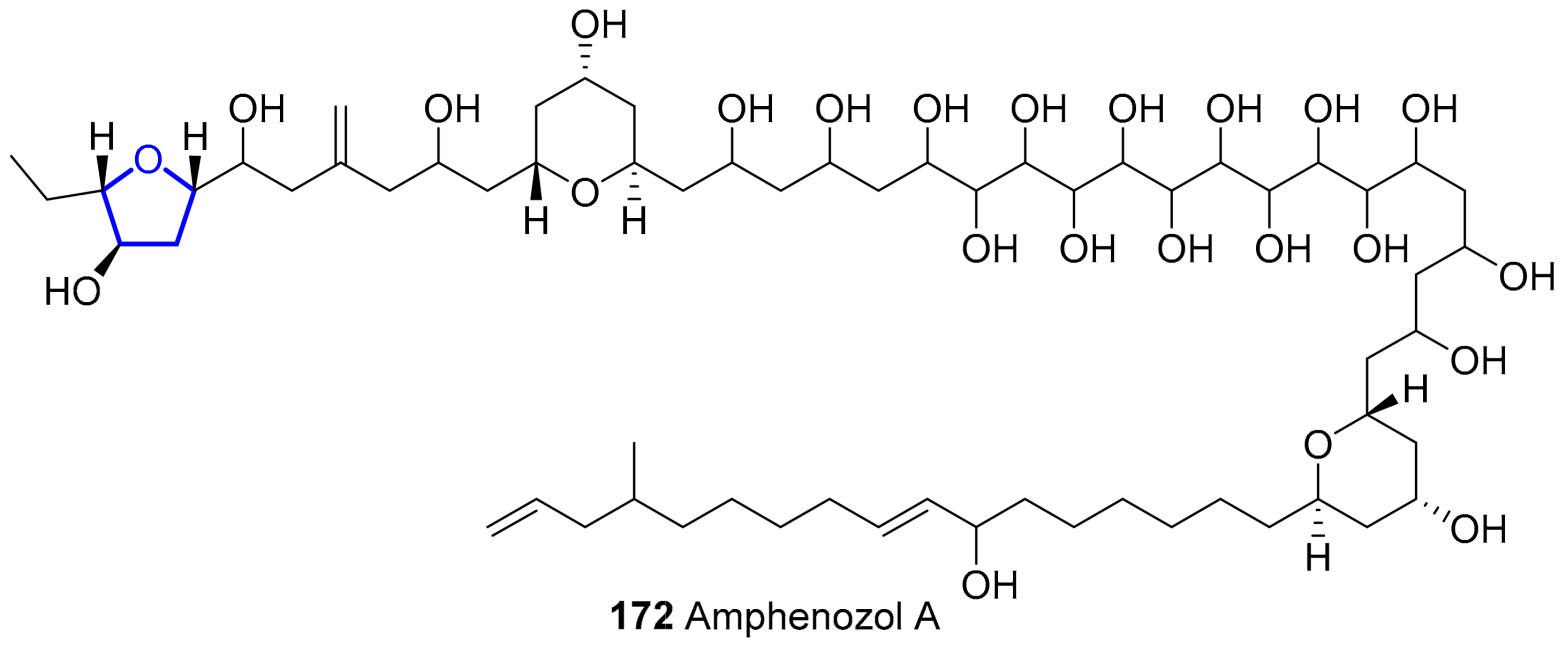
Structure of amphenozol A.

The dinoflagelatte *Amphidinium* sp. has been extensively studied in order to discover and isolate new compounds bearing interesting biological properties, such as macrolides, amphidinolides or polyhydroxyl compounds. In 2006, a novel polyhydroxyl metabolite was isolated, amphenozol A (**172**) [115], the structure of which consists of a C60 linear aliphatic chain with two tetrahydropyran rings, one tetrahydrofuran ring, and twenty-one hydroxyl groups (Figure 25). Its structure was established by NMR experiments. Additionally, the biological analysis showed a modest inhibitory activity against DNA polymerase α.

### 2.6. Bycyclic

#### 2.6.1. Asperpentenone

**Figure 26:**
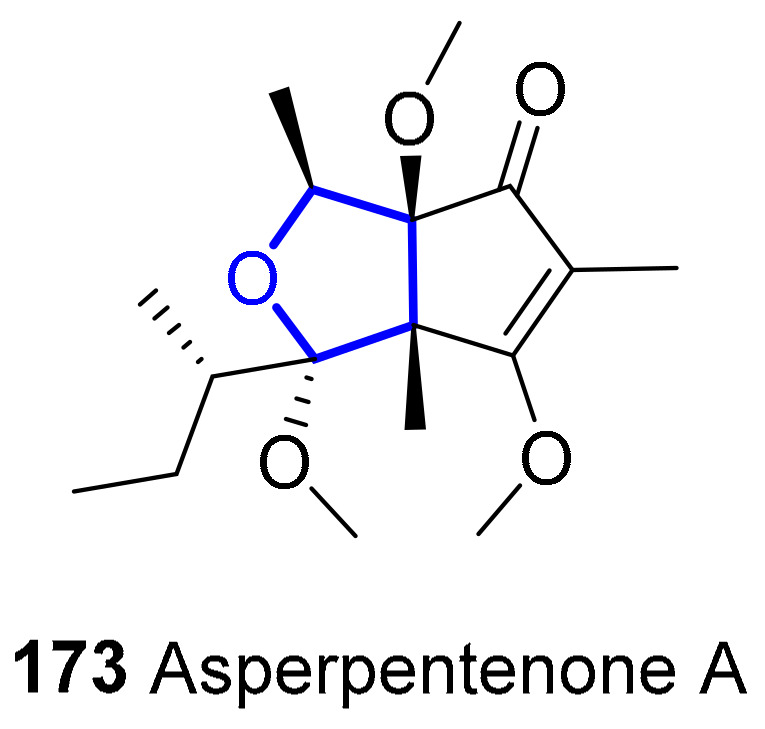
Structure of asperpentenone A.

Recently, J. Wang et al. isolated a novel polyketide, asperpentenone A, from the fungus *Aspergillus* sp. [116]. Its structure, elucidated by nuclear magnetic resonance techniques and X-ray diffraction, contains a cyclopentenone-tetrahydrofuran moiety (Figure 26). So far, no biological activity of asperpentenone A has been described.

#### 2.6.2. Plakortones

**Figure 27:**
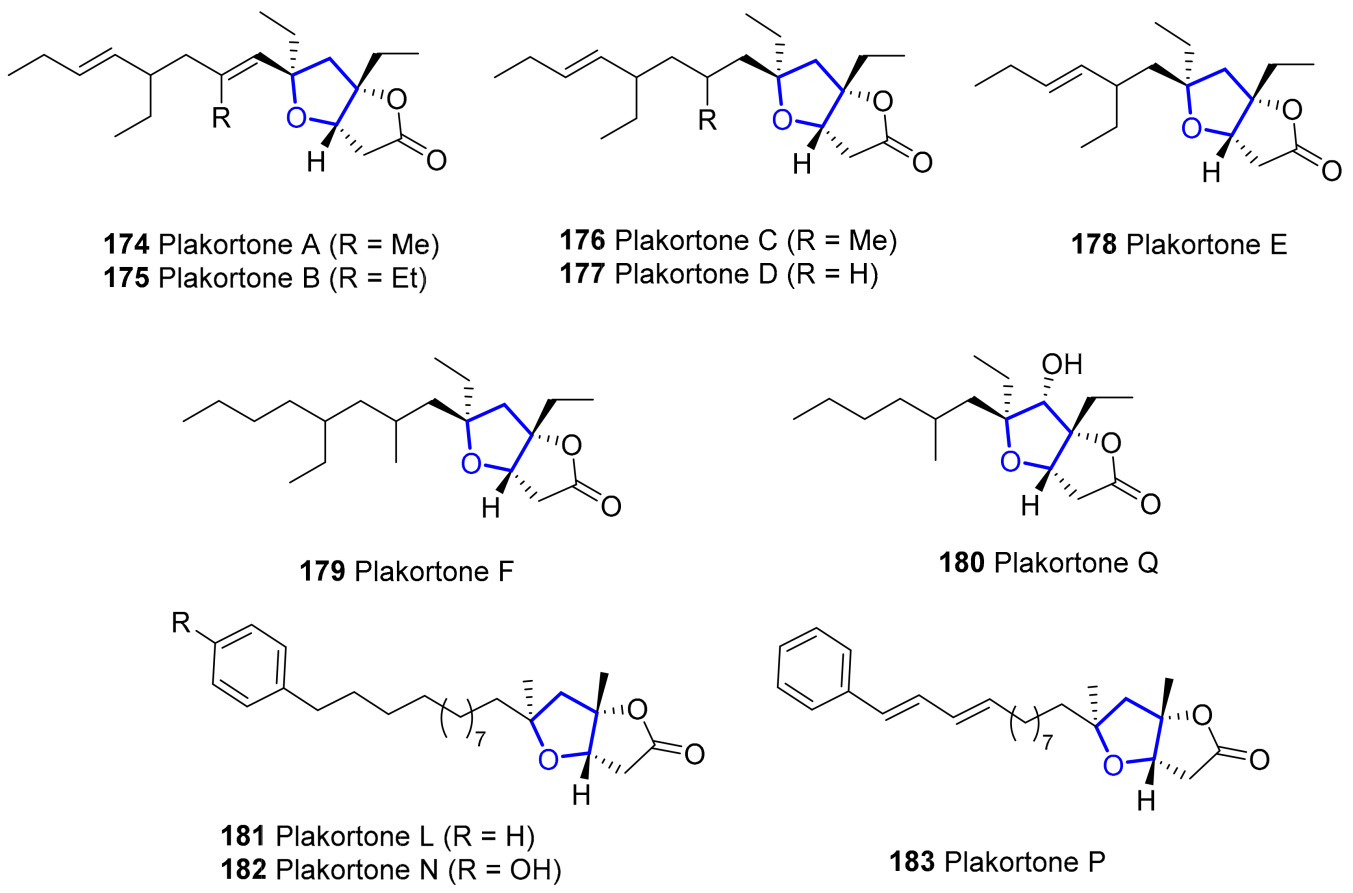
Structure of plakortones.

Ten lactone metabolites, known as plakortones (**174**–**183**), have been isolated from the marine sponge of the genus *Plakortis*. Plakortones belong to a big family of oxygenated polyketide metabolites, the structures of which contain a bicyclic system composed of a tetrahydrofuran fused to a γ-butyrolactone ring (Figure 27). Some of them have been discovered and characterized during the 20th century [117,118], while plakortone L, N, P, and Q were recently isolated [119,120].

**Scheme 33:**
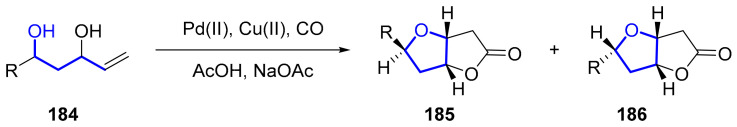
Synthesis of plakortone core.

Since their discovery, numerous studies can be found in the literature with different approaches towards the synthesis of these natural products, their epimers [121] or analogues [122]. A palladium-(II)-mediated hydroxycyclization-carbonylation-lactonization cascade [123] was the general methodology applied to obtain the plakortone core in some cases [122,124,125]. There, from diols **184**, the byclyclic diastereomers **185** or **186** can be accessed (Scheme 33).

**Scheme 34:**
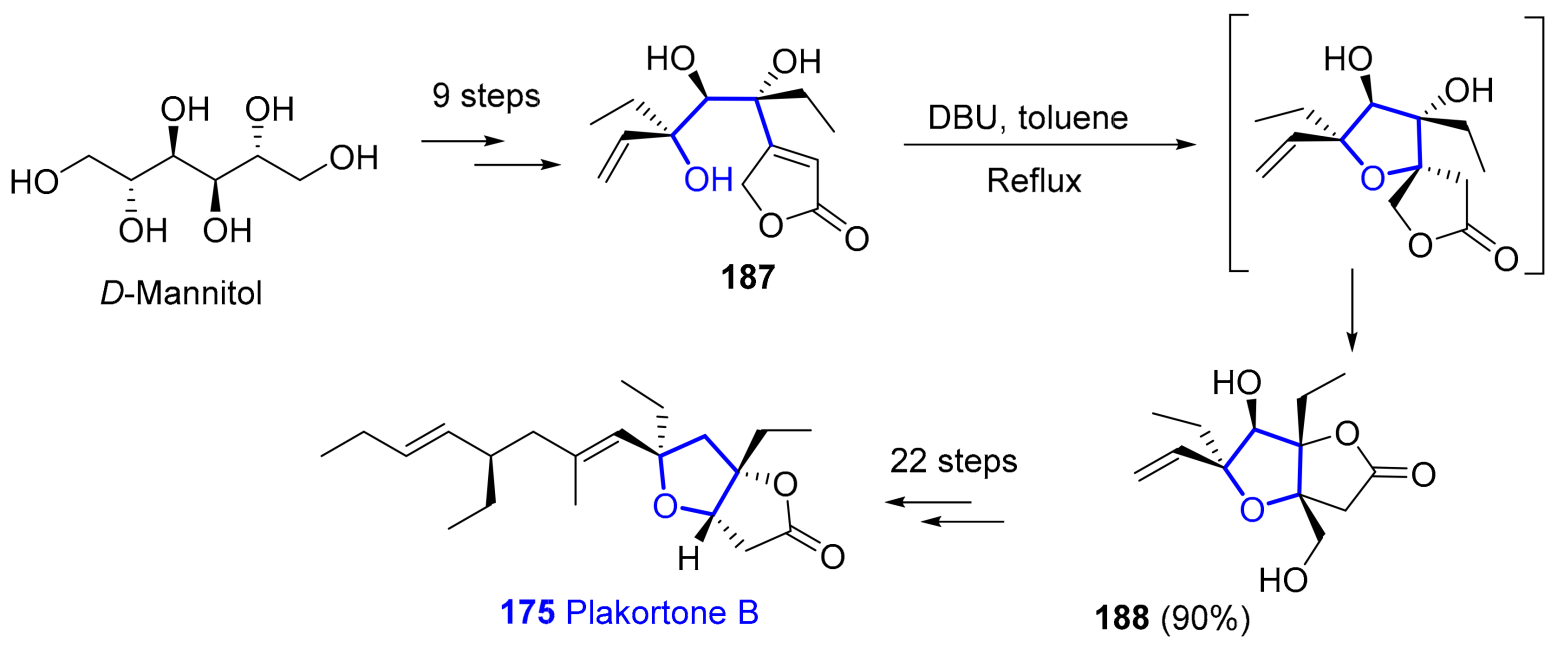
Total synthesis of plakortone B.

The synthesis of the four possible diastereoisomers and comparison with the natural product allowed Wong and co-workers to determine the absolute configuration of the four stereocenters of plakortone B [126]. Thus, retrosynthetic analysis revealed butenolide **187** as the potential precursor of the bicyclic lactone. Formation of butenolide required ten steps from D-mannitol. The bicyclic framework **188** was directly formed with a 90% yield by the reaction of butenolide with 1,5-diazabicyclo[5.4.0]undec-5-ene (DBU), in a domino Michael addition followed by transesterification. Total synthesis of plakortone B was achieved in 22 further steps with a low overall yield (<1%) (Scheme 34).

**Scheme 35:**
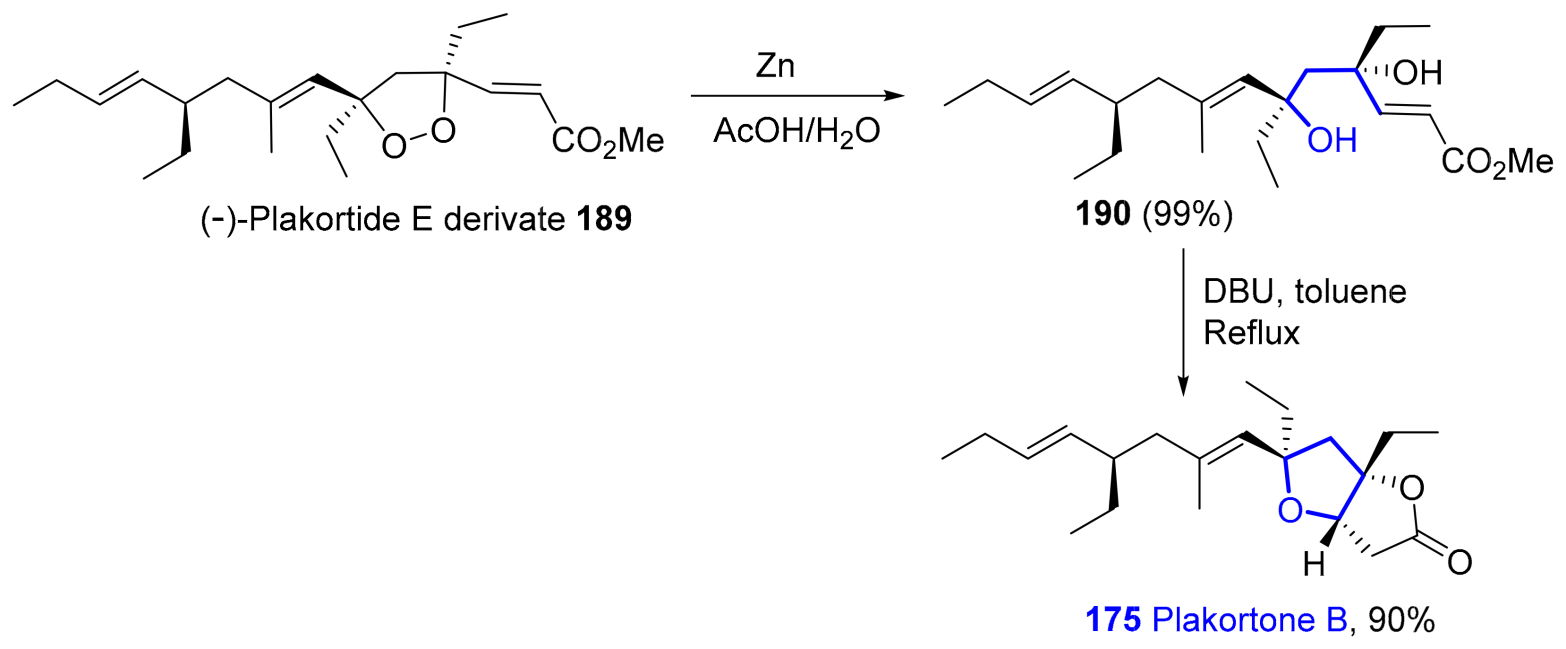
Synthesis of plakortone B via plakortide E derivative.

Another biomimetic approach converted plakortide E derivative **189** into plakortone B [127]. Treatment of **189** with zinc in acetic acid broke the peroxy O-O bond and provided diol intermediate **190**. Further intramolecular oxa-Michael addition/lactonization cascade reaction afforded the desired product with high yield (90%) (Scheme 35).

**Scheme 36:**
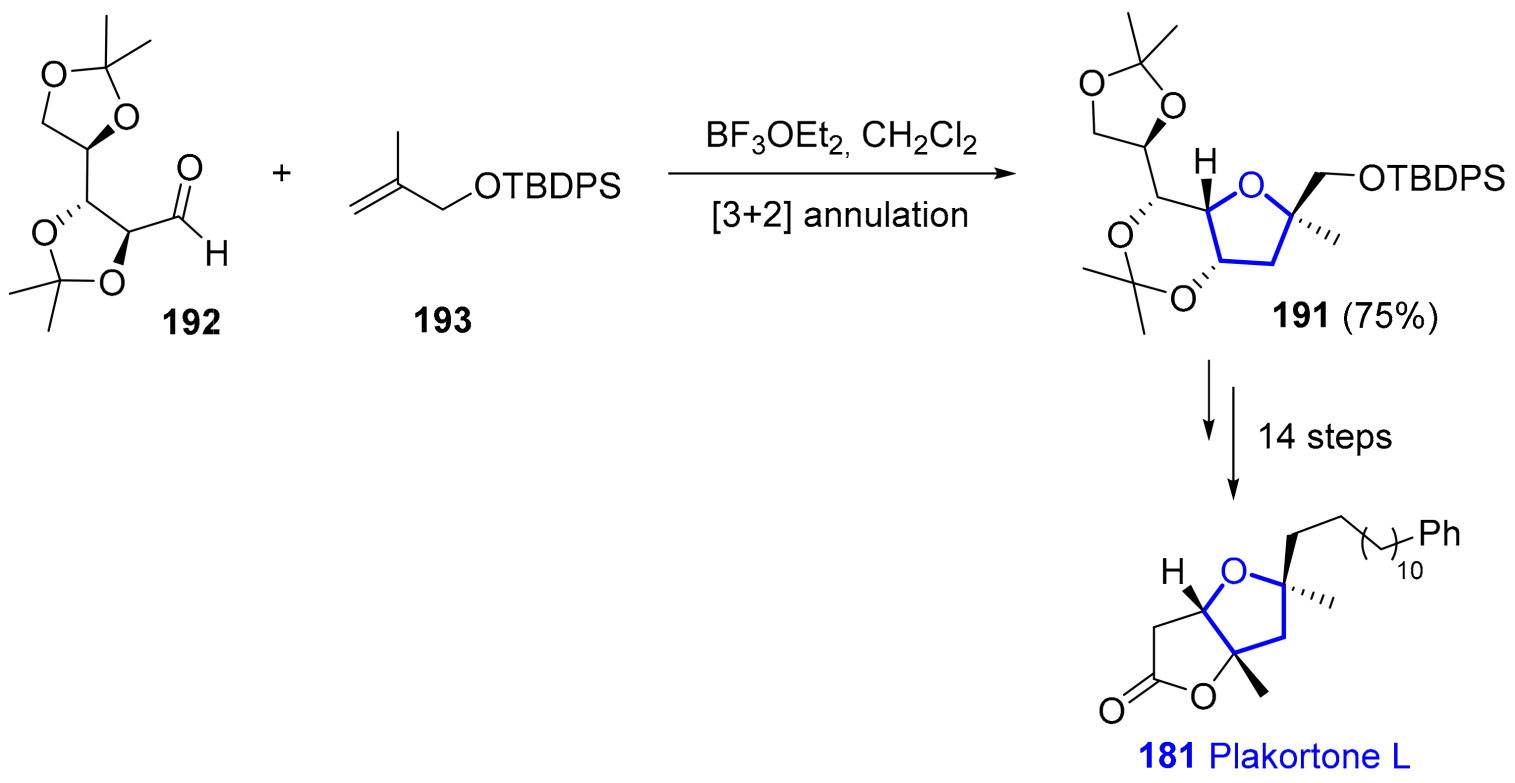
Synthesis of plakortone L.

In 2014, the total synthesis of plakortone L was also reported [128]. In this case, the strategy to obtain the tetrahydrofuran ring was a [3+2] annulation. Thus, the tetrahydrofuranyl ring **191** was obtained by the reaction of isopropylidene-protected D-arabinose **192** and protected methallyl alcohol **193** in the presence of BF_3_·OEt_2_. Fourteen further steps were required to accomplish the total synthesis of plakortone L with 6% overall yield (Scheme 36).

In terms of their biological activity, plakortones A-D belong to the class of activators of cardiac sarcoplasmic reticulum Ca^2+^-pumping ATPase at micromolar concentrations, specially plakortone D [117]. Plakortones B-F exhibit in vitro cytotoxic activity on a murine fibrosarcome cell line [122]. However, plakortone Q, being the only member of the family which contains a hydroxyl group in the ring system, is not active against any of the tested tumor cell lines [129].

## 3. Conclusions

Polyketides are a class of marine natural products with immense structural and biological diversity. They have interesting biological properties that sometimes make them potential drug candidates, such as in eribulin mesylate. Here, organic synthesis in general, and total synthesis in particular still play an important dual role. First, it is the final way of determining the correct structure of a product. Even though, nowadays, there are cutting-edge NMR techniques available, we still see many cases of structure misassignment in marine bioactive products. Secondly, the scarcity of some marine products makes them unavailable for research, and synthesis provides sufficient amounts for proper biological studies. The tetrahydrofuran motif is common among marine polyketides; thus, the development of new methodologies for the synthesis of tetrahydrofurans is a current need.

## Abbreviations

Acac: acetylacetone; AIBN: azobisisobutyronitrile; CAB: (acyloxy)borane; CAN: ceric ammonium nitrate; CSA: camphorsulfonic acid; CuTC: copper(I) thiophene-2-carboxylate; DBU: 1,8-diazabicyclo[5.4.0]undec-7-ene; dba: dibenzylideneacetone; DDQ: 2,3-dichloro-5,6-dicyano-*p*-benzoquinone; (+)-DET: diethyl tartrate; (DHQD)_2_PHAL: hydroquinidine 1,4-phthalazinediyl diether; DMAP: 4-dimethylaminopyridine; DMI: 1,3-dimethyl-2-imidazolidinone; DMF: dimethylformamide; DMI: 1,3-dimethyl-2-imidazolidinone; DMP: Dess-Martin periodinane; DMPA: dimethylol propionic acid; DMPM: 3,4-dimethoxybenzyl; DMSO: dimethyl sulphoxide; KHMDS: potassium bis(trimethylsilyl)amide; LiHDMS: lithium bis(trimethylsilyl)amide; *m*CPBA: meta-chloroperoxybenzoic acid; MNBA: 2-methyl-6-nitrobenzoic anhydride; MOM: methoxymethyl; Ms: methanesulfonyl; NMP: *n*-methylpyrrolidone; PMB: *p*-methoxybenzyl; PPTS: pyridinium *p*-toluenesulfonate; PT: 1-phenyltetrazol-5-yl; TASF: tris(dimethylamino)sulfonium difluorotrimethylsilicate; TBAF: tetrabutylammonium fluoride; TBCO: 2,4,4,6-tetrabromo-2,5-cyclohexadienone; TBDPS: *tert*-butyldiphenylsilyl; TES: triethylsilyl; TFA: trifluoroacetic acid; TIPS: triisopropylsilyl; TMS: trimethylsilyl; Tr: triphenyl methyl; Ts: tosyl.
